# GIP attenuates neuronal oxidative stress by regulating glucose uptake in spinal cord injury of rat

**DOI:** 10.1111/cns.14806

**Published:** 2024-06-17

**Authors:** Beibei Guo, Mengwei Qi, Xiaoqian Luo, Longyu Guo, Man Xu, Yufang Zhang, Zhen Li, Mingxuan Li, Ronghua Wu, Tuchen Guan, Mei Liu, Yan Liu

**Affiliations:** ^1^ Key Laboratory of Neuroregeneration of Jiangsu and Ministry of Education, Co‐innovation Center of Neuroregeneration, Medical School Nantong University Nantong China; ^2^ Key Laboratory of Neuroregeneration of Jiangsu and Ministry of Education, Co‐innovation Center of Neuroregeneration, NMPA Key Laboratory for Research and Evaluation of Tissue Engineering Technology Products Nantong University Nantong China

**Keywords:** Akt, GIP, glucose transporter, HIF‐1α, oxidative stress, spinal cord injury

## Abstract

**Aim:**

Glucose‐dependent insulinotropic polypeptide (GIP) is a ligand of glucose‐dependent insulinotropic polypeptide receptor (GIPR) that plays an important role in the digestive system. In recent years, GIP has been regarded as a hormone‐like peptide to regulate the local metabolic environment. In this study, we investigated the antioxidant role of GIP on the neuron and explored the possible mechanism.

**Methods:**

Cell counting Kit‐8 (CCK‐8) was used to measure cell survival. TdT‐mediated dUTP Nick‐End Labeling (TUNEL) was used to detect apoptosis in vitro and in vivo. Reactive oxygen species (ROS) levels were probed with 2', 7'‐Dichloro dihydrofluorescein diacetate (DCFH‐DA), and glucose intake was detected with 2‐NBDG. Immunofluorescence staining and western blot were used to evaluate the protein level in cells and tissues. Hematoxylin‐eosin (HE) staining, immunofluorescence staining and tract‐tracing were used to observe the morphology of the injured spinal cord. Basso‐Beattie‐Bresnahan (BBB) assay was used to evaluate functional recovery after spinal cord injury.

**Results:**

GIP reduced the ROS level and protected cells from apoptosis in cultured neurons and injured spinal cord. GIP facilitated wound healing and functional recovery of the injured spinal cord. GIP significantly improved the glucose uptake of cultured neurons. Meanwhile, inhibition of glucose uptake significantly attenuated the antioxidant effect of GIP. GIP increased glucose transporter 3 (GLUT3) expression via up‐regulating the level of hypoxia‐inducible factor 1α (HIF‐1α) in an Akt‐dependent manner.

**Conclusion:**

GIP increases GLUT3 expression and promotes glucose intake in neurons, which exerts an antioxidant effect and protects neuronal cells from oxidative stress both in vitro and in vivo.

## Graphical abstract

GIP increases GLUT3 expression and facilitates glucose uptake via Akt/HIF‐1α axis, which protects neurons from oxidative stress and promotes functional recovery of spinal cord injury.

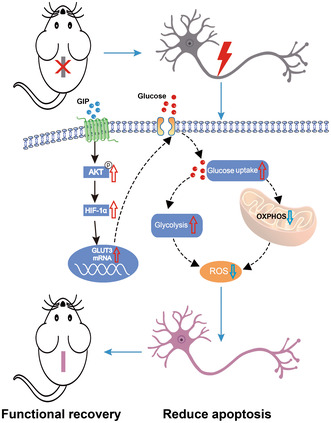

## 1 INTRODUCTION

Spinal cord injury always leads to ischemia and hypoxia within the injured area, which is accompanied with a sharp increase of ROS.^1^, ^2^ ROS is mainly produced by mitochondria, which are reciprocally the target of harmful effects of ROS.^3^ ROS could interact with proteins to trigger an apoptotic cascade, which leads to the death of neuronal cells. Therefore, inhibition of oxidative stress is a key issue in protecting injured tissue and reserving neural function. The injury‐induced ischemia and hypoxia also reduce glucose utilization in neural tissues. Then, metabolic stress is another obstacle for neural tissues after injury. The approach to attenuate oxidative stress and energy shortage will promise a favorable output for nerve injury.^4^

GIP is an intestinal hormone that belongs to the glucagon polypeptide superfamily. It consists of 42 amino acids and is secreted by intestinal endocrine cells K cells under the stimulation of food intake. GIP is well‐known for its ability to stimulate insulin secretion that is essential for maintaining glucose homeostasis.^5^ Meanwhile, a growing body of evidence indicates that GIPR is expressed in many tissues other than the pancreas islet, which proposes alternative physiological effects of GIP in an insulin‐independent manner. The previous studies clearly suggest that GIPR signaling plays a role in regulating osteogenesis. GIP increases cAMP and intracellular calcium levels in osteoblasts and improves type I collagen expression during osteogenesis.^6^

GIP/GIPR is also implicated in peripheral inflammation in a tissue/cell‐specific manner. Peripheral in vivo infusion of GIP could increase proinflammatory cytokines in adipocytes.^7^ GIP and GIPR expression have also been detected in the nervous system.^8^ In our previous study, we demonstrated that GIP/GIPR could significantly promote axon growth of cortical neuron^9^ and facilitate Schwann cell migration after periphery injury.^10^ Moreover, GIP was reported to show neuroprotective effects in animal models of neurodegenerative disease,^11^, ^12^ while the underlying mechanism is elusive yet.

In this study, we revealed the antioxidant effect of GIP during nerve injury. GIP/GIPR regulates systemic levels of blood glucose via modulating insulin secretion. The neuronal expression of GIPR proposed a possibility of whether GIP/GIPR could directly regulate neuronal glucose metabolism and exert a protective effect. Then we investigated the effect of GIP/GIPR signaling on oxidative and metabolic stress during spinal cord injury and explored the mechanism of the protective effect of GIP.

## 2 MATERIALS AND METHODS

### 2.1 Animal

Sprague–Dawley rat embryos at 18 days (E18) and adult rats (200 ± 20 g) were obtained from the Laboratory Animal Center of Nantong University (Nantong, China) and kept in a temperature and humidity‐controlled environment with a light/dark cycle of 12/12 h, food and water freely available. The rats were sacrificed by CO_2_ inhalation. All biological sample acquisition procedures were reviewed and approved by the Institutional Animal Care and Use Committee of Nantong University. All animal surgeries were conducted in accordance with Institutional Animal Care guidelines, as well as with the National Institutes of Health (Bethesda, MD) guidelines.

### 2.2 Primary E18 neuron culture

Cortical neurons were obtained from Sprague–Dawley rats at E18. The bilateral cerebral cortex was placed in a culture dish containing ice‐cold Hank's buffer (Cat. C0218; Beyotime, Shanghai, China), followed by the removal of the meninges. The cortex tissues were treated with 0.25% trypsin–EDTA for 12 min at 37°C, then gently dropped through a sterile 38‐μm Nitex mesh (BD Falcon, San Jose, CA, USA). The cells were resuspended in DMEM/F‐12 (Cat. 10‐092‐CV; Thermo Fisher Scientific, USA) supplemented with 10% FBS, 1% penicillin–streptomycin (Cat. C0222; Beyotime), and 0.5 mM glutamine (Cat. C0212; Beyotime), and plated into the Petri dishes which coated with 0.1 mg/mL Poly‐L‐Lysine (PLL Cat. P4832, Sigma) to allow incubation in a humidified atmosphere of 5% CO_2_ at 37°C for 6 h. Then, the culture medium was replaced by Neurobasal medium (Cat. 2,110,349; Gibco) supplemented with 2% NeuroCult™ SM1 Neuronal Supplement (Cat. 05711; Stem Cell), 1% penicillin–streptomycin, and 0.5 mM glutamine.

### 2.3 RNA isolation and quantitative real‐time PCR

Total RNA was isolated from cultured cortical neurons using the RaPure Total RNA Micro Kit (Cat. R4012‐2; Magen Biotech, Guangzhou, China) according to the manufacturer's instructions. Briefly, neurons were lysed with lysis buffer and mixed with an equal volume of RNA binding buffer. RNA samples were added to the spin column, centrifuged, and washed with RNase‐free water. The concentrations of isolated RNA samples were determined using a NanoDrop spectrophotometer (Thermo Fisher Scientific, USA). Complementary DNA (cDNA) was synthesized using the OminScript RT Kit (Qiagen, Germantown, USA), and quantitative real‐time polymerase chain reaction (qRT‐PCR) was performed using the DyNAmo Flash SYBR Green qPCR Kit (Thermo Fisher Scientific, USA) following the manufacturer's instructions. Two micrograms of cDNA per well were used to detect the relative mRNA levels. Relative mRNA expression levels were calculated according to the 2^−ΔΔCT^ method. 18 s was used as an internal control. The sequences of the primers used in this study are listed in Table 1.

**TABLE 1:** Primer sequences used in this study.

Name	Sense (5′‐3′)	Antisense (5′‐3′)
*18 s*	GGACACGGACAGGATTGACA	CAATCTCGGGTGGCTGAAC
*Glut3*	TCAACCGCTTTGGCAGACGCA	AGGCGGCCCAGGATCAGCAT
*Hk1*	GAAATCTTAACCCGCTTGG	TGTGCCCTTGTTGTCCC
*Pfkfb3*	GTCTGTGACGACCCTA	CTTCTGCGGAGTTGC
*Pkm2*	TCTGGAGGCTGTTCGC	GGGTCGCTGGTAATGG
*Ldha*	AAAGTCCAAGATGGCAGCCC	GCTCATCAGCCAAGTCCTTCA
*Sod1*	CGAGCAGAAGGCAAGC	GCCCAGGTCTCCAACA
*Sod2*	AGCCTCCCTGACCTGC	CCCCGCCATTGAACTT
*Sod3*	CCAGCGGGTTGTAGTGT	CCAGCGGGTTGTAGTGT
*Cat*	TGAAGCAGTGGAAGGAGC	CATCTTGAACGAGGAGGG
*Gpx*	CGGGACTACACCGAAAT	CCGCAGGAAGGTAAAGA

### 2.4 Spinal cord injury

Adult male Sprague–Dawley rats weighing 200 ± 20 g were provided by the Laboratory Animal Center of Nantong University (Nantong, China). Animal surgeries were performed according to Institutional Animal Care and National Institutes of Health (Bethesda, MD, USA) guidelines, and all procedures were approved by the Administration Committee of Experimental Animals of Jiangsu Province. Anesthesia was conducted with isoflurane in oxygenated air provided by a small animal anesthesia machine (RWD Life Science, Jiangsu, China). Sprague–Dawley rats were placed on a temperature‐maintaining device in a prone position. Surgeries were performed by the same person to ensure injury consistency. A T10 lateral hemisection surgical procedure based on the protocol used in our previous study was utilized.^13^ After anesthesia and disinfection with 75% alcohol, an incision was made in the dorsal midline skin. The subcutaneous tissue extending at T10 and the muscle and tissue overlying the spinal column were bluntly dissected to expose the T10 laminae. A hemisection was then carefully created at T10 on the right side with an ophthalmic iris knife. The muscle layers were sutured, and the skin was secured with wound clips. After surgery, 100 μL gentamicin‐saline was applied at the injury site for anti‐infection.

### 2.5 Tract tracing of propriospinal neurons

The tracing procedure of spinal cord proprioceptive neurons was performed as follows, with T8 laminectomy performed without touching the spinal cord. The tracer virus (pAAV2/9‐syn‐mCherry‐3FLAG; OBIO), was injected into the spinal cord at a rate of 0.15 μL/min using a special fine glass needle with a microinjection pump. The injection method was as follows: before the spinal cord was semi‐transversed, one injection point was set at 0.2 mm from the central canal of the spinal cord on each side, and 0.4 μL was injected at each point; 0.2 μL was injected 0.7 mm below the dura mater, and then 0.2 μL was slowly lifted up to 0.5 mm. To prevent reflux and virus spread, the needle was suspended for 2 min after injection. So as not to obstruct the view, T10 lateral hemisection surgical was not performed until all microinjections were completed.

### 2.6 Drug treatment

For cortical neurons, after culturing 36 h, the primary cultured cortical neurons were treated with 100 μM H_2_O_2_ for 3 h, then the H_2_O_2_ was removed and 100 nM GIP was added for 24 h. To investigate the molecular pathway, inhibitor for AKT signal pathway (Mk2206, 20 μM, Cat. HY‐10358; MCE Co., Monmouth Junction, NJ, USA) and inhibitor for HIF‐1α signal pathway (KC7F2, 20 μM, Cat. S7946; Selleck) and 2‐Deoxy‐D‐glucose (Cat. HY‐13966; MCE Co., Monmouth Junction, NJ, USA) was added into cell culture for further assays.

In the rat spinal cord injury model, 10 μM D‐Ala2‐GIP was injected in situ near the injury site with a glass electrode needle, and a total of three sites were injected, 1 μL for each site. Then D‐Ala2‐GIP was injected into the tail vein at the dose of 50 nmol/kg every 2 days, and about 500 μL was injected into each rat according to the body weight. The changes of the levels of reactive oxygen species and proteins were detected 3 days after the injury. Hematoxylin‐eosin staining was used to observe the morphology of spinal cord tissue at 3 and 14 days after spinal cord injury. BBB functional scores were performed on the hind limbs of rats at 1, 3, 7, 10, and 14 days after spinal cord injury to assess the functional recovery of the hind limbs.

### 2.7 In situ spinal cord GIP injection

During the T10 hemisection of the rat spinal cord, GIP was injected according to the need of the experiment. A glass micropipette (with a tip diameter of about 20 μm) was mounted on a microfeeding pump, and 3 μL of GIP was aspirated at a time, while the control group was injected with the same volume of saline; after exposing the spinal cord, a dry cotton ball was used to remove the excess blood and tissue fluid to ensure a wide field of vision; the injection site was determined, and the spine of the rat was fixed using the Rodent Stereotactic Positioning Device (RSPD). Move the glass micropipette to the dura mater, and set the Z‐axis to 0. In this experiment, there were three injection points for GIP, located on the opposite, above, and below side of the hemisection injury; 10 μM GIP was injected into the spinal cord at T10 with a speed of 0.2 μL/min, and the same injection point was divided into three injection depths; 0.3 μL of GIP was injected into the spinal cord at a distance of 1.2 mm below the dura mater, and the needle was slowly lifted to 0.2 mm below the dura mater, and then the needle was injected into the spinal cord at a speed of 1.2 mm below the dura mater. Inject 0.3 μL at 1.2 mm below the dura mater, slowly lift the needle to 0.9 mm and inject 0.3 μL, and finally position the injection depth to 0.6 mm below the dura mater and inject 0.3 μL, and then stop the needle for 1–2 min after the injection.

### 2.8 Peptide

The GIP and GIP analogs D‐Ala^2−^GIP (Peptide Purity: 96.91%) were obtained from the Nanjing Yuanpeptide Biological Technology (Nanjing, China). The purity of the peptide was confirmed by reversed‐phase HPLC and characterized using matrix‐assisted laser desorption/ionization time of flight (MALDI‐TOF) mass spectrometry. Peptide sequence of the GIP analogs D‐Ala^2^GIP: Y‐(D‐Ala)‐EGTFISDYSIAMDKIRQQDFVNWLLAQK.

### 2.9 Cell viability, apoptosis analysis

Cell counting Kit‐8 (CCK‐8) was used to measure cell survival according to the manufacturer's protocol. Cultured neurons were inoculated into 96‐well plates with 3 × 10^4^ per well for 36 h. Cells were treated with the indicated drug and incubated with 10 μL CCK‐8 solution (Cat. A311; Vazyme) per well 2 h before the specified timepoint. Optical density (OD) at 450 nm was measured using a miniature tablet reader (Synergy 2; Bio Tek, USA).

To assess cell apoptosis, samples were washed with cold PBS and fixed with precooled 4% paraformaldehyde at 4°C for 25 min, then stained using an apoptosis analysis kit (Cat. A112; Vazyme) according to manufacturer's instructions. The number of apoptotic cells was evaluated by BD FACSCalibur (BD Biosciences, USA).

### 2.10 Detection of ROS levels

#### 2.10.1 ROS assay for cell

ROS levels were probed with DCFH‐DA produced by China Maokang Biological Company. The samples were cleaned twice with PBS and the DCFH‐DA (Cat. MX4801‐1KIT; Maokang) solution of 2.5 μM was added to the cells. Then, the cells were incubated at 37°C for 20 min. The cells were washed three times with serum‐free cell culture solution and photographed with Leica fluorescence microscope.

#### 2.10.2 ROS assay for tissue

ROS levels were probed with DCFH‐DA produced by China Bestbio Company (Cat. BB‐470535). Specific steps are: Prepare 10 μm thick unfixed frozen sections to be tested; at room temperature, carefully add 200 μL of cleaning solution, spread over the entire section surface, and let it stand for 5–10 min; carefully remove the cleaning solution; add 100 μL of staining solution drop by drop, and incubate at 37°C in an incubator, protected from light, for 20–60 min; remove the staining solution; wash the sections with PBS for 2–3 times; seal the sections with coverslips or glycerol; and detect the sections with fluorescence microscope.

### 2.11 Glucose uptake assay

Utilizing 2‐NBDG (Cat. GC10289, Glpbio), a glucose uptake assay was performed as described previously.^14^ 2‐NBDG solution was diluted to 100 μM with glucose‐free neurobasal. Cells were seeded in plates. After treatment with drugs, cortical neurons were treated with 2‐NBDG diluted solution for 15–120 minat 37°C. The cells were observed with a DMi8 microscope (Leica Microsystems, Wetzlar, Germany). The fluorescence intensity was analyzed by ImageJ.

### 2.12 Immunofluorescence assay

Immunofluorescence (IF) assays were performed as described previously.^15^ Cultured neurons were fixed with a buffer containing 4% paraformaldehyde, 0.2% glutaraldehyde, 1× PHEM (60 mM PIPES, 25 mM HEPES, 10 mM EGTA, and 2 mM MgSO_4_), and 0.1% Triton X‐100 for 20 min. Cultures were then washed with PBS three times and blocked with 10% goat serum containing 10 mg/mL bovine serum albumin for 60 min at 37°C. Cultures were incubated with primary antibodies (rabbit anti‐Tuj1, 1:1000, Cat. ab18207, Abcam; mouse anti‐Glut3, 1:500, Cat. Sc‐74,399, Santa; mouse anti‐Glut1, 1:500, Cat. ab40084, Abcam; rabbit anti‐Glut4, 1:500, Cat. ab654, Abcam.) overnight at 4°C. After rewarming for 30 min the next day, the cultures were rinsed with PBS and then incubated with the corresponding species‐specific fluorescence‐conjugated secondary antibodies (488‐conjugated goat anti‐rabbit IgG, 1:400; cy3‐conjugated goat anti‐mouse IgG, 1:400; Jackson ImmunoResearch, West Grove, PA, USA) at room temperature for 2 h. The cells were counterstained with DAPI stain (1:2500, Cat. D9542, Merck, Germany) at room temperature for 15 min. The cells were then washed with PBS and mounted in an anti‐fade mounting medium.

For immunohistochemistry, rats were perfused with 4% paraformaldehyde, and perilesional spinal cord tissues measuring 1.5 cm were collected and cryoprotected in 30% sucrose. Then, tissue sections (14 μm) were prepared by cryostat sectioning and treated with a blocking buffer (0.4% BSA, 5% goat serum, and 0.3% Triton‐X 100 in PBS) for 120 min at room temperature. Primary antibodies against glial fibrillary acidic protein GFAP (1:1000, Cat. ab4676; Abcom), GAP43 (1:200, Cat. 16,971‐1‐AP; Proteintech), Tuj1 (1:800, Cat. 801,201; BioLegend), mcherry (1:600, Cat. ab205402; Abcam), and chondroitin sulfate proteoglycan CSPG (1:400, Cat. C8035; Sigma) were incubated overnight at 4°C in a blocking buffer. After rinsing with PBS, the corresponding secondary antibodies were incubated for 120 min at room temperature. All the tissues were counterstained with DAPI. The tissues were washed and mounted. Fluorescence images were obtained using a fluorescence microscope (Zeiss).

For fluorescence intensity analysis, images were transformed to 16‐bit TIFF, and analyzed in ImageJ for mean fluorescence intensity. For neurite length analysis, images were obtained at 20× using a Zeiss microscope and analyzed in IPP for length using length analysis.

### 2.13 Western Blotting

Cells or tissues were lysed to extract total proteins, which were further quantified using BCA reagents (Cat. P0011; Beyotime). Total protein samples (20 μg) were separated by SDS‐PAGE and then transferred onto PVDF membranes. After blocking with 5% skim milk, the membranes were incubated overnight at 4°C with the following primary antibodies (rabbit anti‐P‐Akt, 1:1000, Cat. 13,038, CST; rabbit anti‐Akt, 1:1000, Cat. 4685 s, CST; mouse anti‐Glut3, 1:1000, Cat. sc‐74,399, Santa; rabbit anti‐AMPK, 1:1000, Cat. 5832 s, CST; rabbit anti‐P‐AMPK, 1:1000, Cat. 2535 s, CST; rabbit anti‐PKA, 1:1000 Cat. 4782, CST; rabbit anti‐P‐PKA, 1:1000, Cat. 5661, CST; rabbit anti‐mTOR, 1:1000, Cat. 2983, CST; rabbit anti‐P‐mTOR, 1:1000, Cat. 2976, CST; rabbit anti‐BAX, 1:1000, Cat. 2772 T, CST; rabbit anti‐Bcl2, 1:1000, Cat. 26,593‐1‐AP, Proteintech; mouse anti‐β‐Actin, 1:1000, Cat. 66,009‐1‐lg, Proteintech; rabbit anti‐HIF‐1α, Cat. Ab179483, Abcam). After washing with TBST and 0.1% Tween 20, membranes were incubated with horseradish peroxidase‐conjugated secondary anti‐rabbit or anti‐mouse antibodies at room temperature for 2 h. Blots were covered with ECL (Cat. 180–5001; Tanon). The membrane was then covered in the chemical ECL A/B mix and visualized with X‐ray films or a chemiluminescence image analysis system (Tanon 5200), and analyzed using Multi Gauge software (FujiFilm Corp. Life Science Division, Tokyo, Japan). β‐Actin was used as a loading control.

### 2.14 Oxidative damage of neurons

The neurons were cultured in Petri dishes coated with poly‐lysine. After 36 h, the basal medium was replaced with Neurobasal medium containing 100 μM H_2_O_2_ for 3 h, the medium containing H_2_O_2_ was discarded and the neurons were cultured for a further 24 h.

### 2.15 Hematoxylin‐eosin staining

Hematoxylin‐eosin staining experiment was conducted using the kit provided by Beyotime Biotechnology Company. Remove frozen slices from the −80°C refrigerator and rewarm them at room temperature for 15 min. Wash the slice with water for 3 min and remove OCT embedding agent. Then frozen slices were incubated with hematoxylin for 7 min. Discard hematoxylin and wash slices with dd H_2_O for 30 min. Then stain with eosin for 10 s. Discard eosin and put the slices into the staining tank as follows: 95% ethanol (2 s), 95% ethanol (2 s), 100% ethanol (2 s), and 100% ethanol (1 min). In the fume hood, put the frozen slices into the following glass dyeing jars: xylene I (1 min), xylene II (1 min), xylene III (1 min). Then blow and dry the slices in the fume hood. After that, use sealing tablets (neutral resin: xylene = 7:3) to take pictures.

### 2.16 Statistical analysis

The data were presented as mean ± standard error (SE), and all data were tested for normal distribution. The Student's *t‐*test was used to compare the means between two groups, whereas one‐way ANOVA was used to compare the means among three or more groups. The BBB scores were analyzed using two‐way ANOVA and post hoc Bonferroni's test. Prior to statistical analyses, the data sets for each group were tested for normality of distribution using the Kolmogorov–Smirnov test. Values of *p* < 0.05 were considered statistically significant. Data analysis was performed using GraphPad Prism version 8.0.

## 3 RESULTS

### 3.1 Effect of GIP on oxidative stress of cultured neurons in vitro

**FIGURE 1:**
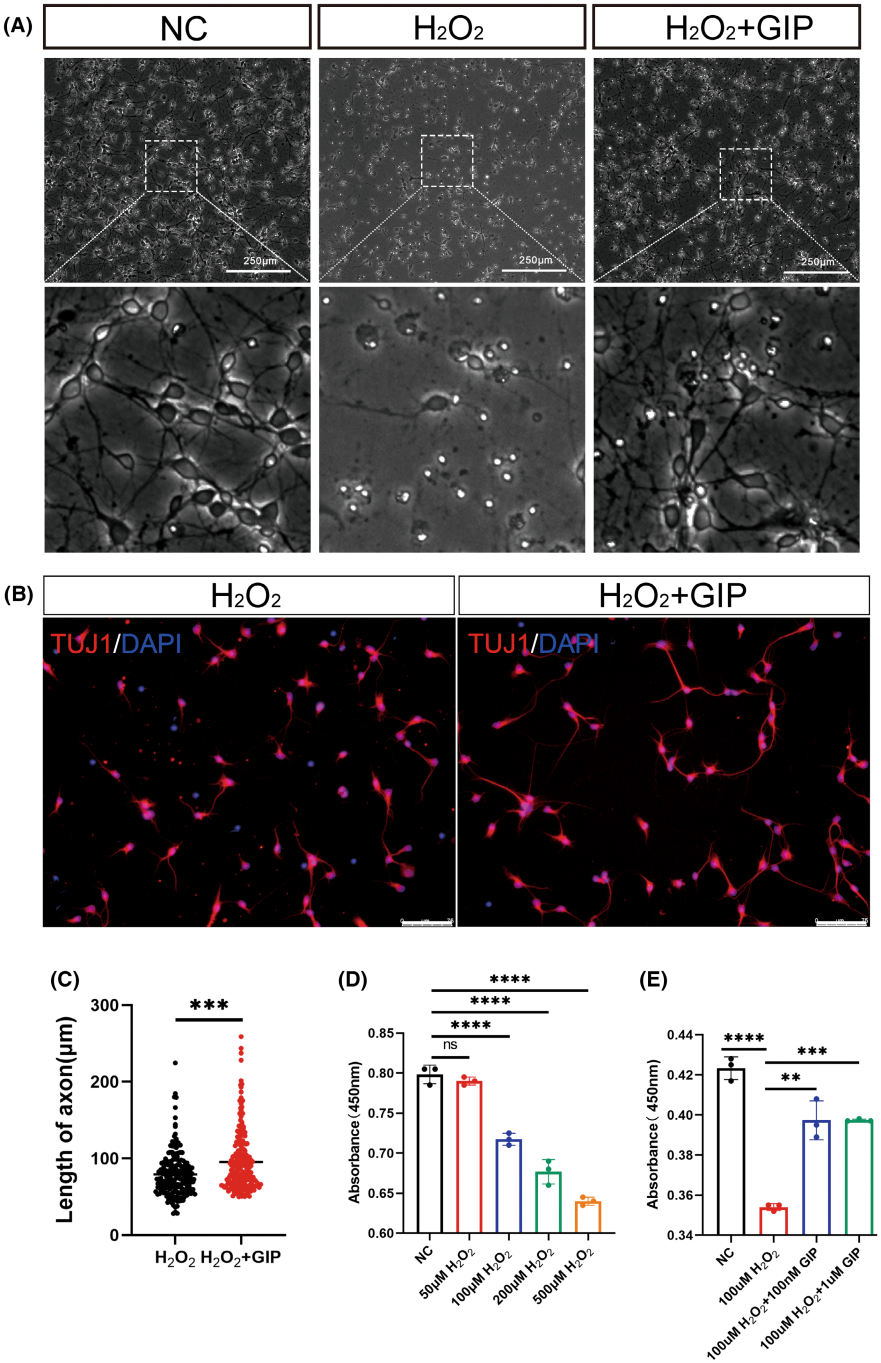
GIP restored the cell viability of primary cultured E18 cortical neurons after oxidative damage. (A) Representative images of neurons after 3 h of H_2_O_2_ injury and 24 h of GIP treatment. The enlarged views of dotted boxes were shown in the down panel. Scale Bar = 250 μm. (B) Representative images of neurons stained with anti‐TUJ1 (red), DAPI (blue) after 3 h of H_2_O_2_ injury and 24 h of GIP treatment. Scale Bar = 75 μm. (C) GIP increased the length of the axon. The data were shown as mean ± SE, and were analyzed by Student's *t*‐test, *n* = 200–220 neurons for each group per test, *N* = 3 (cells were from rats interpedently). ***p < 0.001. (D) Cell viability was evaluated by CCK8 assay after treatment with different concentrations of H_2_O_2_. The data were shown as mean ± SE, and were analyzed by one‐way ANOVA, *n* = 3, ns represents no statistical difference, ****p < 0.0001. (E) GIP attenuates the detrimental effect of H_2_O_2_ on cultured neurons. Neurons were treated with 100 μM H_2_O_2_ for 3 h and treated with 100 nM or 1 μM GIP for 24 h. The data were shown as mean ± SE, and were analyzed by one‐way ANOVA, *n* = 3. **p < 0.01, ***p < 0.001, ****p < 0.0001.

Excess ROS is a major cause of injury and death in neurological diseases for the reason that neurons are susceptible to oxidative damage than other cells.^16^, ^17^ We cultured E18 cortical neurons and treated them with H_2_O_2_ of different concentrations (50, 100, 200, and 500 μM). Morphological analysis of neurons revealed a decrease in cell density and axon length following oxidative damage (100 μM H_2_O_2_) to neurons, all of which were significantly ameliorated by GIP stimulation (Figure 1A–C). Axon length significantly increased after GIP treatment (Figure 1B,C). The results of CCK‐8 assay showed that the cell viability decreased significantly after the treatment of 100 μM H_2_O_2_ for neurons, and the administration of 200 and 500 μM H_2_O_2_ caused more cell death (Figure 1D). Then the treatment of 100 μM H_2_O_2_ was applied for the following experiments. The administration of GIP significantly improved the cell viability of neurons upon H_2_O_2_ treatment as shown in Figure 1E, which proposed a protective effect of GIP.

**FIGURE 2:**
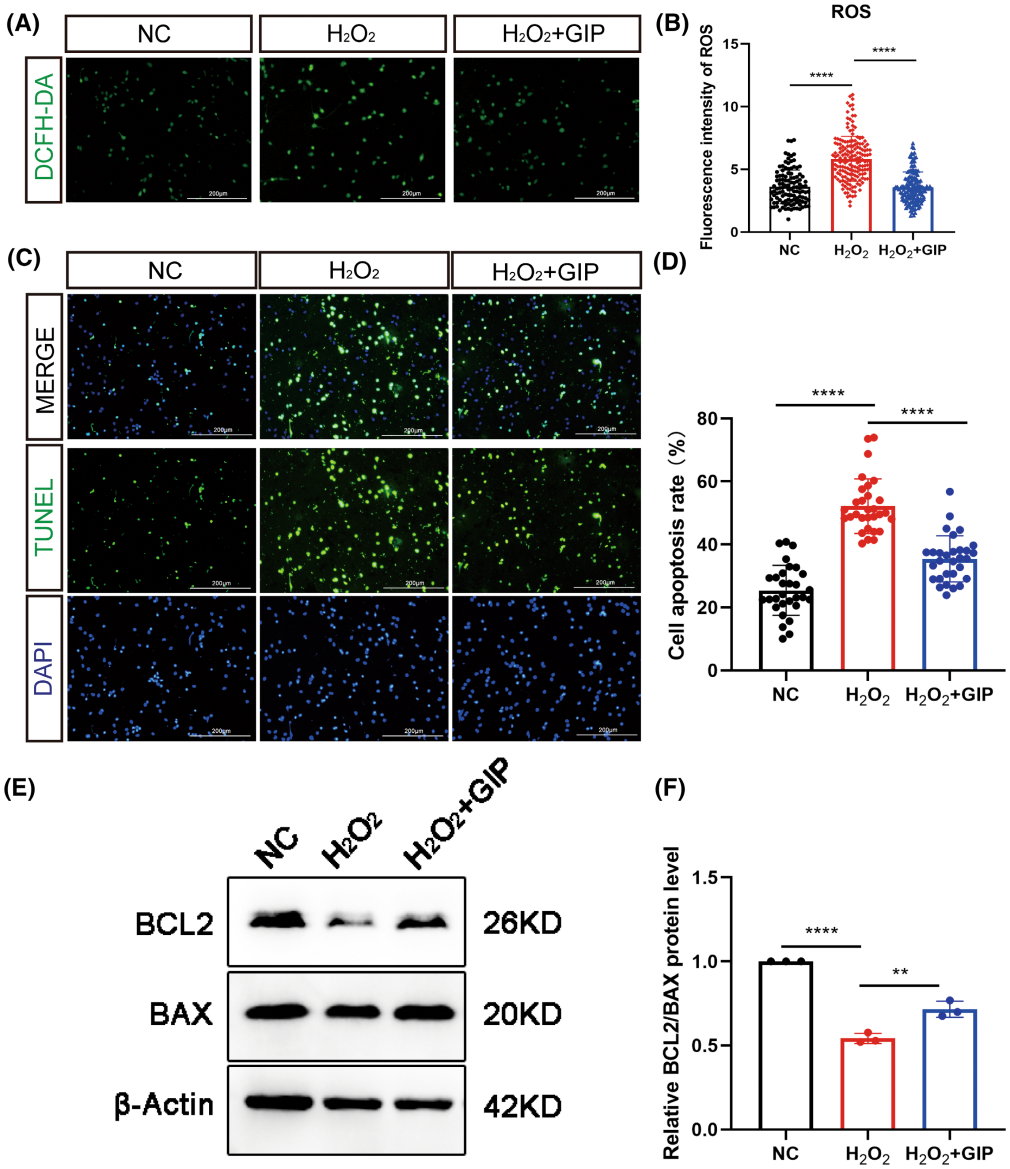
Effect of GIP on the ROS level in neurons under oxidative stress. (A, B) GIP decreased ROS level after H_2_O_2_ treatment. ROS signals were probed by DCFH‐DA. Scale Bar = 200 μm. *n* = 100–150 neurons for each group per test, *N* = 3 (cells were from rats interpedently). The data were shown as mean ± SE, and were analyzed by one‐way ANOVA, ****p < 0.0001. (C, D) GIP decreased the number of apoptotic cells. The cell apoptosis was determined by TUNEL assay. *n* = 30 for each group per test, *N* = 3. The data were shown as mean ± SE, and were analyzed by one‐way ANOVO, ***p < 0.001, ****p < 0.0001. Scale Bar = 200 μm. (E, F) GIP increased the ratio of BCL2/BAX protein after treatment of H_2_O_2_. The data were normalized by negative ctrl (NC) group. *n* = 3. The data were shown as mean ± SE, and were analyzed by one‐way ANOVA, **p < 0.01, ****p < 0.0001.

Under normal conditions, small amounts of ROS are produced within cells, and these ROS play an important role in cellular signaling and can be cleared by antioxidant defense systems.^18^ Under oxidative stress, the excessive ROS exceeds the buffering capacity of the antioxidant system, which can lead to cell damage and death. In this study, we observed the effect of GIP on ROS levels in neurons, which increased sharply after oxidative injury and decreased significantly after GIP treatment (Figure 2A,B). These results showed that GIP reduced intracellular ROS production after oxidative damage. We next examined the effect of GIP on the apoptosis of cultured neurons, as shown in Figure 2C,D, with a significant increase in the number of apoptotic cells in the H_2_O_2_ group and a significant decrease in the number of apoptotic neurons after GIP treatment. GIP could decrease the number of apoptotic cells after oxidative damage. We further used western blot to identify the expression levels of apoptosis‐related markers Bcl‐2 and BAX in neurons. The ratio of these two proteins indicated that GIP treatment attenuated H_2_O_2_‐induced apoptosis (Figure 2E,F).

In addition, we examined the mRNA level of ROS‐related genes (SOD1, SOD2, SOD3, GPX, and CAT) in cultured neurons and in the model of spinal cord injury upon GIP treatment. However, we did not observe significant changes between the control and GIP group (Figure S1). These data indicated the GIP did not regulate the transcription of these ROS‐related genes, and might exert the antioxidant effect via alternative mechanisms, such as metabolic regulation.

### 3.2 Protective effect of GIP on spinal cord injury model of rat

Ischemia and hypoxia after traumatic spinal cord injury always lead to a sharp increase in ROS level,^19^ which could result in direct cell damage and the development of secondary injury.^20^, ^21^, ^22^ Therefore, we sought to determine whether GIP could reduce ROS levels and show protective effects in the model of rat spinal cord injury, surgery for SCI can be referenced in the schematic in Figure S2.

**FIGURE 3:**
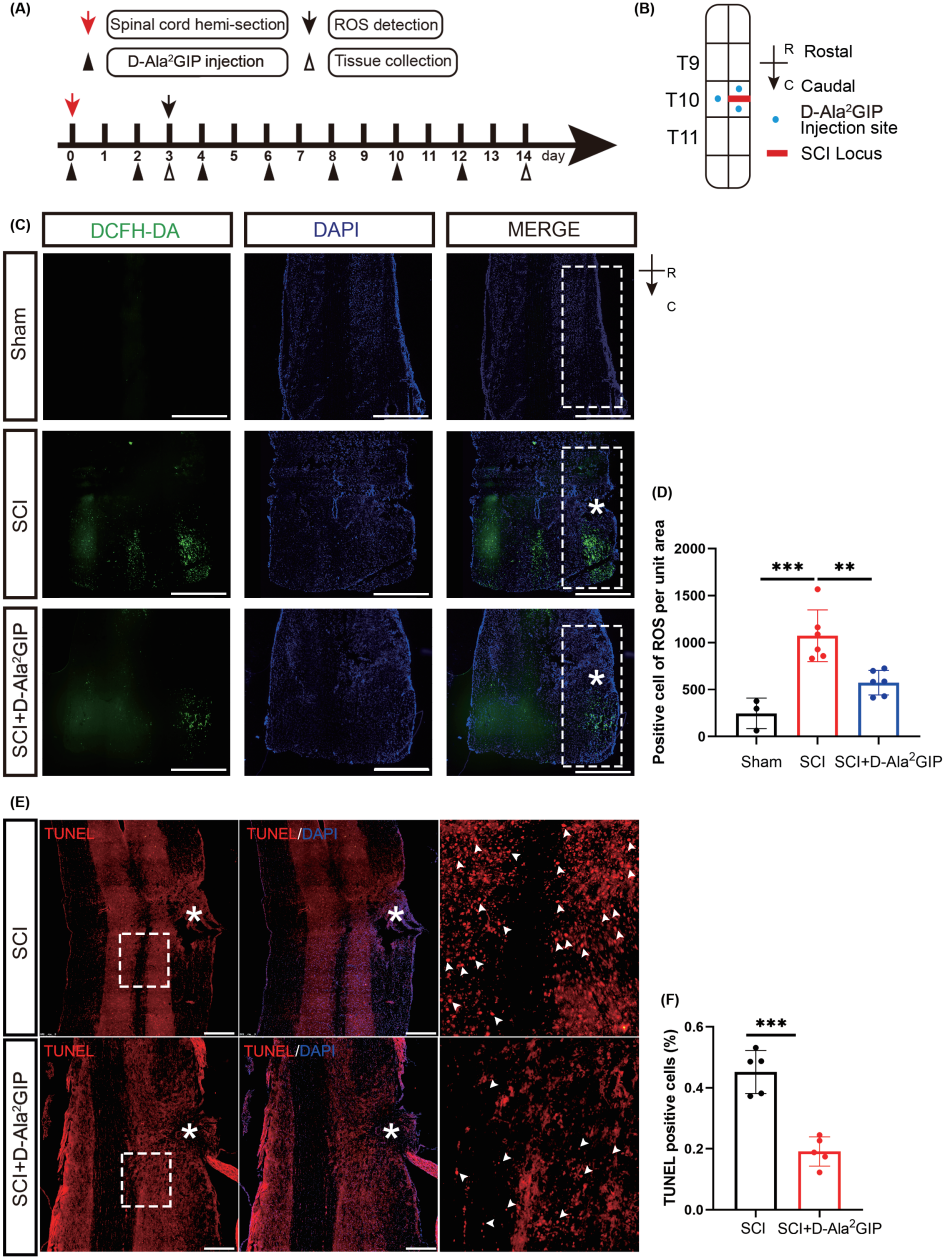
Effect of GIP on oxidative stress in rats with spinal cord hemisection injury. (A) Timeline of spinal cord hemisection injury, D‐Ala^2^GIP administration, and tissue collection. (B) Model of GIP injection in spinal cord injury. Spinal cord hemisection was conducted at T10. For saline and D‐Ala^2^GIP treatments, each rat was injected with 3 μL saline or D‐Ala^2^GIP at three sites. R: Rostral, C: Caudal. (C, D) D‐Ala^2^GIP decreased ROS level after spinal cord hemisection injury for 3 days. The white “*” shows the injury sites and the white box shows the area for statistics of ROS signal. Scale Bar = 500 μm. Statistical results of ROS‐positive cells were shown. *n* = 3 for sham group, *n* = 6 for SCI group and SCI + GIP group. The data were shown as mean ± SE, and were analyzed by one‐way ANOVA, **p < 0.01, ***p < 0.001. (E, F) D‐Ala^2^GIP decreased the number of apoptotic cells after spinal cord hemisection injury for 14 days. The white “*” shows the injury sites, the white arrows are representative TNUEL‐positive signals, and the images on the right are magnified views of the boxed area. Scale Bar = 250 μm, Statistical result of TUNEL mean fluorescence intensity after spinal cord hemisection injury for 14 days. *n* = 5 for each group. The data were shown as mean ± SE, and were analyzed by Student's *t*‐test, ***p < 0.001.

GIP is a short peptide that is easy to be degraded by dipeptidyl peptidase‐4 (DPP‐4) in vivo.^23^ Then, we used the modified GIP (D‐Ala^2^GIP) that substituted the left‐handed Ala with right‐handed Ala for the experiment of spinal cord injury, which acts as a GIPR agonist with a long half‐life in vivo.^24^ The spinal cord hemisection injury was conducted and tail intravenous injection of D‐Ala^2^GIP was performed at a dose of 50 nmol/kg (the schematic diagram of drug administration and spinal cord injury is shown in Figure 3A,B). The results (Figure 3C,D) showed that GIP significantly reduced the DCFH‐DA probed ROS signals in the injury site on day 3 after spinal cord injury. To observe the effect of GIP on the level of apoptosis in vivo, TUNEL assay was conducted and the results showed (Figure 3E,F) that apoptotic cells were significantly reduced in the GIP‐treated group after spinal cord injury. In addition, we also detected the protein levels of BCL2/BAX to show the effect of GIP treatment on apoptosis. The results showed that GIP significantly increased the ratio of BCL2/BAX that was consistent with the data of TUNEL assay (Figure S3). These data suggest that GIP can reduce oxidative stress and apoptosis after spinal cord hemisection injury in vivo.

### 3.3 GIP promotes recovery of spinal cord injury

**FIGURE 4:**
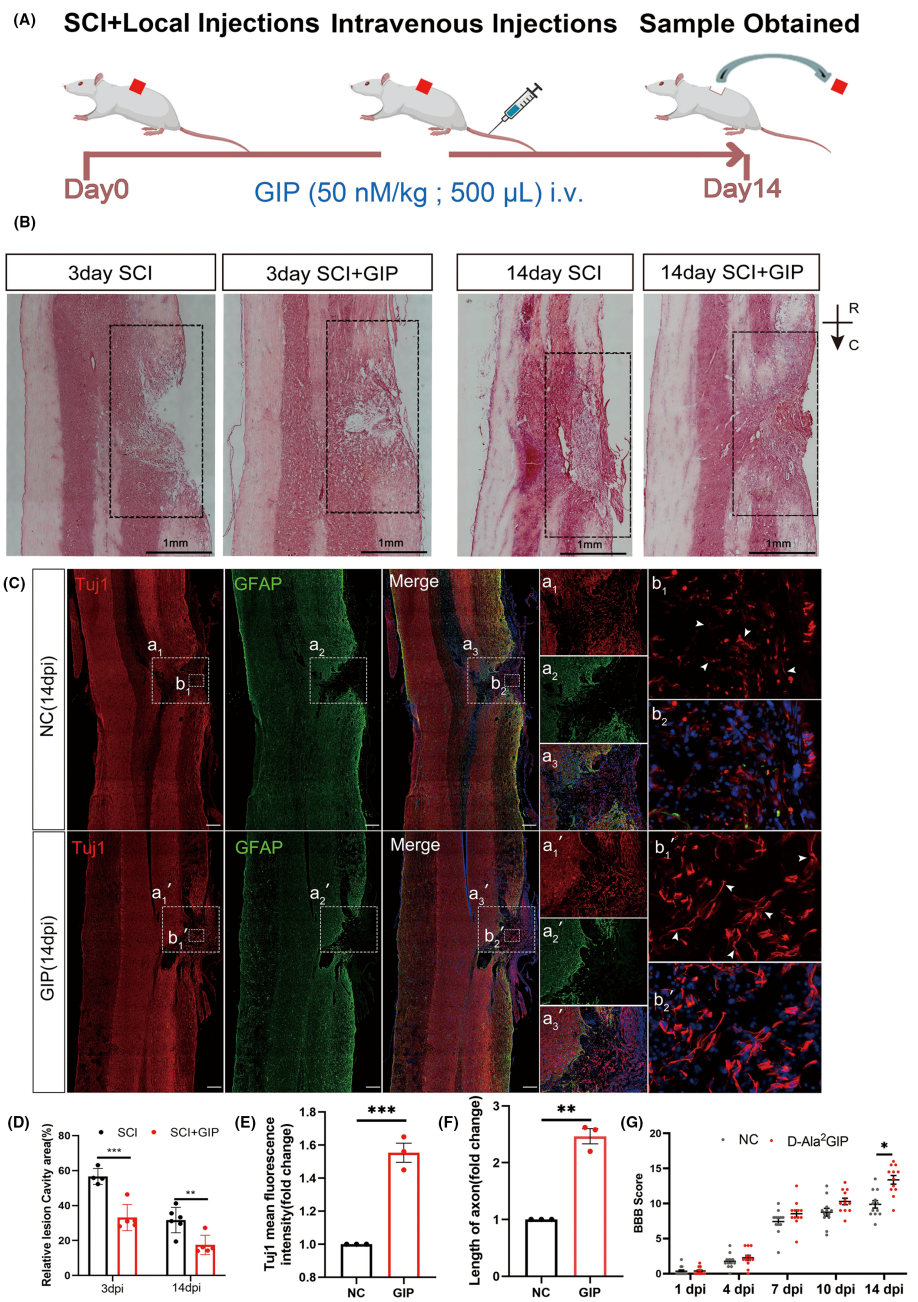
Effect of D‐Ala^2^GIP on locomotor function and histomorphology recovery in spinal cord injured rats. (A) Schematic diagram of experiment. D‐Ala^2^GIP was injected at a dose of 50 nmol/kg. (B, D) GIP reduced the area of the cavity of the injured spinal cord. Representative HE staining and statistical result. The box showed the area of injury. Scale Bar = 1 mm; R: Rostal, C: Caudal. *n* = 6 for each group. The data were shown as mean ± SE, and were analyzed by two‐way ANOVA post hoc Bonferroni's test, **p < 0.01, ***p < 0.001. (C, E, F) GIP increased the number neurites grown into injured area. Representative immunostaining staining and statistical results of Tuj1 (in red), GFAP (in green) at 14 dpi. Spinal cord was treated with NC or D‐Ala2GIP. Neurons were labeled with Tuj1. The white box shows the injury area and the magnified views are shown in the right panel. Scale Bar = 250 μm. The data were normalized by NC. *n* = 3 for each group. The data were shown as mean ± SE, and were analyzed by Student's *t*‐test, **p < 0.01, ***p < 0.001. (G) BBB scores at 1, 4, 7, 10, and 14 days after spinal cord injury. *n* = 12 for each group. The data were shown as mean ± SE, and were analyzed by two‐way ANOVA post hoc Bonferroni's test, *p < 0.05.

To maintain the concentration of GIP in vivo, we administered intravenous injections to rats every other day after spinal cord injury (Figure 4A). Spinal cord injury results in significant tissue damage featuring with glial scar and tissue cavity. The results of HE staining at 3 dpi and 14 dpi injury are evaluated (Figure 4B,D), the area of injury‐induced cavities was smaller with GIP treatment. Immunofluorescence staining showed more Tuj1 signals in the GIP group than that of the control group at 14 dpi (Figure 4C,E), and the axon length of GIP‐treated neurons entering the injury zone was significantly longer than that of the control group (Figure 4F), which indicated that more neuronal projections were present in injury site after GIP administration. We also evaluated the effect of GIP treatment on functional recovery after spinal cord injury as shown in Figure 4G, the BBB score of SCI rats in the GIP group was higher than those of the control group and showed significant improvement at 14 dpi. The above results demonstrate that GIP can promote morphological and functional recovery after spinal cord injury in rats.

To validate this result, we further performed a virus tracing experiment. The propriospinal neurons were labeled with syn‐mCherry virus before the injury, then the regenerated axon could be traced by mCherry (Figure S4A,B). In addition to TUJ1 immunofluorescence staining, we additionally labeled neurons in the spinal cord using GAP43 (Figure S4C,D). The result proposed that GIP treatment significantly facilitated the axon regeneration of propriospinal neurons.

### 3.4 GIP promotes neuron glucose uptake

Our study revealed the effect of GIP on oxidative stress both in vitro and in vivo. Then we questioned what is the underlying mechanism. Glucose metabolism and oxidative stress are two important metabolic processes in organisms. There is a close relationship between glucose metabolism and oxidative stress. GIP was originally discovered as a glucose‐dependent insulin‐stimulating polypeptide, and also as a dietary‐regulated intestinal polypeptide that is critical for the maintenance of glucose homeostasis. So, can GIP regulate neuronal metabolism and provide metabolic support for nerve injury repair?

**FIGURE 5:**
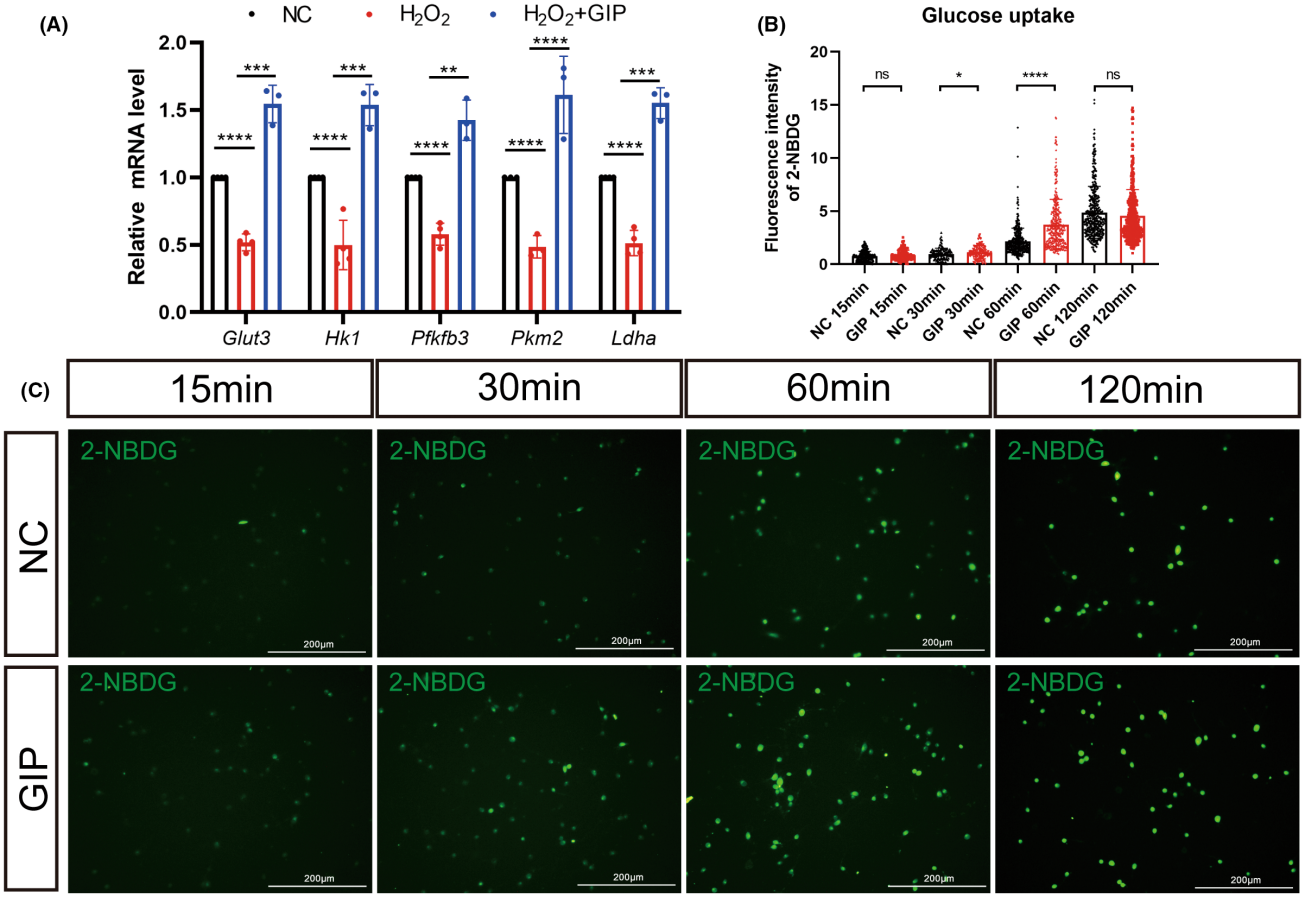
Effect of GIP on glucose uptake in neurons. (A) GIP increased the mRNA level of Glut3, Hk1, Pfkfb3, Pkm2, and Ldha in neurons after H_2_O_2_ treatment. *n* = 3. The data were normalized by NC and shown as mean ± SE, analyzed by one‐way ANOVA, **p < 0.01, ***p < 0.001, ****p < 0.0001. (B, C) GIP augmented glucose uptake in cultured neurons. Statistical results and representative images were shown in B and C. *n* = 150 neurons, *N* = 3 rats, the experiment repeated three times independently. The data were shown as mean ± SE, analyzed by Student's *t*‐test vs. NC 15 min, NC 30 min, NC 60 min, and NC 120 min, ns represents no statistical difference, *p < 0.05, ****p < 0.0001. C: Scale Bar = 200 μm.

We verified the expression of genes for glucose transport and the glycolytic process by qRT‐PCR (Figure 5A). After 24 h of exposure, GIP up‐regulated the expression of GLUT3 and key glycolytic process enzymes in neurons. These results proposed that GIP could modulate glucose intake via regulating glucose transporter. Then, we tested this hypothesis using 2‐NBDG, a fluorescent analog of glucose, as a probe for the detection of glucose uptake in cultured neurons. The cultured neurons in the presence or absence of GIP were incubated with 2‐NBDG for 15, 30, 60, and 120 min to evaluate glucose uptake. We found that the fluorescence intensity of the GIP group was significantly higher than that of the control group after 30 and 60 min incubation of 2‐NBDG (Figure 5B,C). The data showed that GIP can significantly enhance the glucose uptake in neurons.

### 3.5 GIP protects against oxidative damage by regulating glucose transport

**FIGURE 6:**
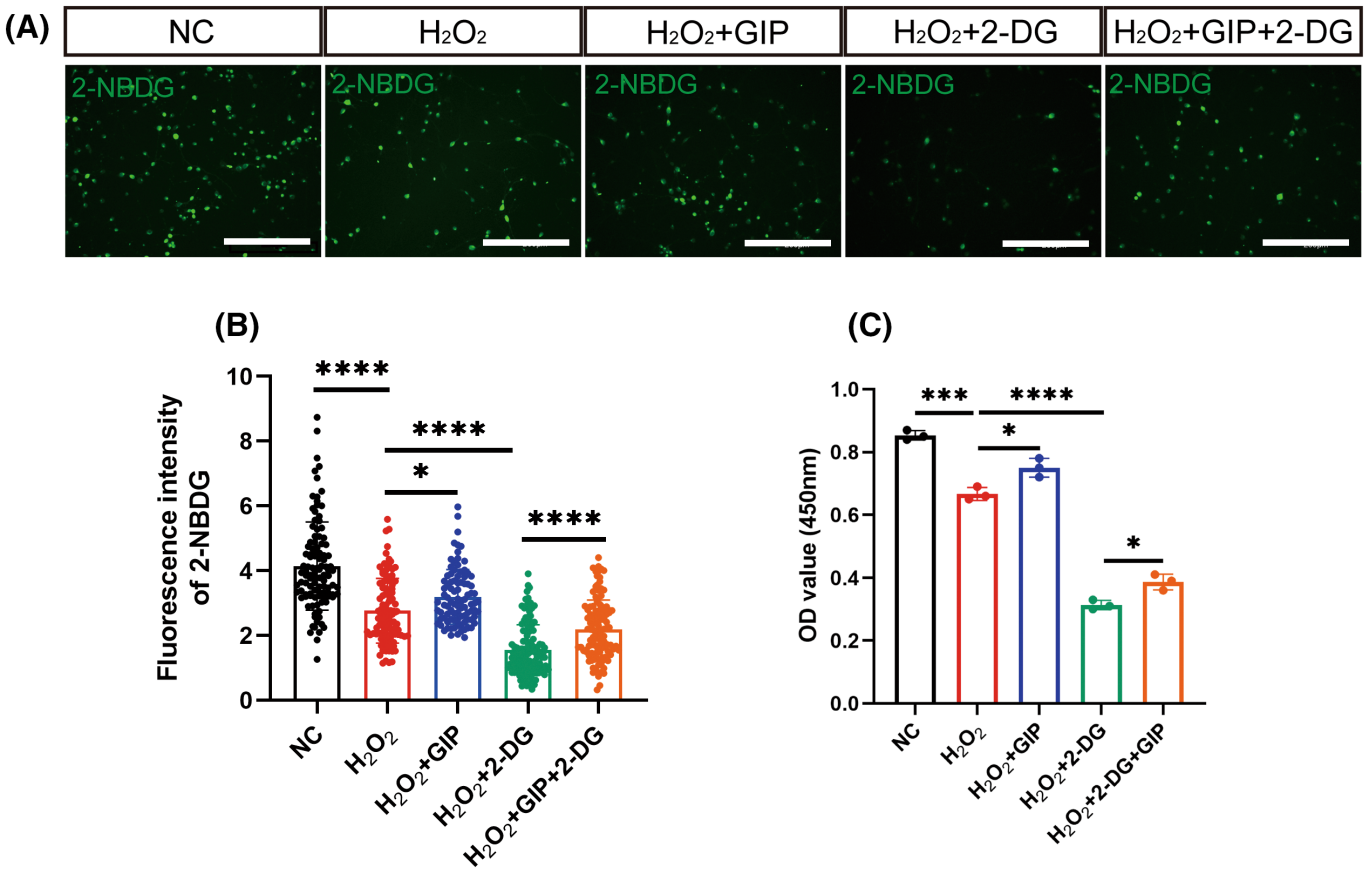
GIP‐protected neurons from oxidative damage by regulating glucose transport. (A) Representative immunostaining result of 2‐NBDG (Green) after GIP and 2‐DG treatment. Scale Bar = 200 μm. (B) Statistical result of 2‐NBDG fluorescence intensity. *n* = 100–150 neurons, *N* = 3 rats. The data were shown as mean ± SE, and were analyzed by one‐way ANOVA, *p < 0.05, ****p < 0.0001. (C) Statistical result of OD value at 450 nm. *n* = 3. The data were shown as mean ± SE, and were analyzed by one‐way ANOVA, *p < 0.05, ***p < 0.001, ****p < 0.0001.

GIP showed protective effect against oxidative stress and facilitated glucose uptake. Then, we asked whether this effect is associated with the glucose transport. Previous studies have reported that 2‐Deoxy‐D‐glucose (2‐DG), a glucose transport inhibitor, is transported across the membrane and rapidly phosphorylated into 2‐deoxy glucose 6‐phosphate, which cannot be further metabolized by glycolytic enzymes, this hinders the transport of glucose.^25^ After GIP treatment of neurons under normal and oxidative conditions, 2‐DG was added, and we found that 2‐DG counteracted the proposed effect of GIP on glucose uptake in neurons (Figure 6A,B). CCK‐8 assay also showed that 2‐DG reversed the enhancing effect of GIP on the viability of oxidatively damaged neurons (Figure 6C). In conclusion, the results suggested that GIP might protect cells from oxidative damage by regulating glucose transport.

### 3.6 GIP increased the expression of the neuron‐specific glucose transporter GLUT3

**FIGURE 7:**
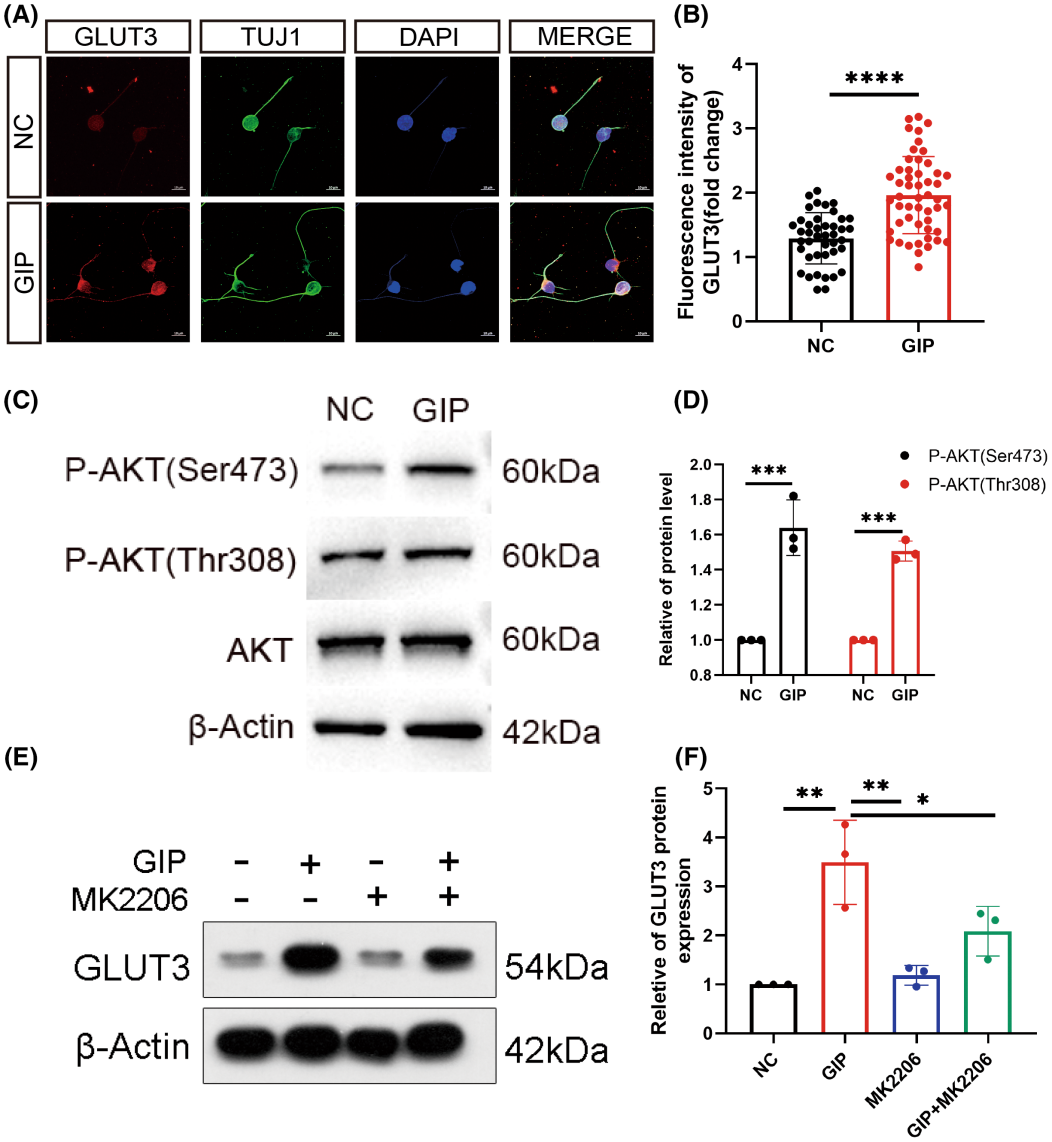
GIP up‐regulated GLUT3 expression via Akt pathway. (A, B) GIP increased GLUT3 level in neurons. Representative immunostaining results and statistical result were shown in A and B. Scale Bar = 10 μm. *n* = 20–50 neurons for each group per test, *N* = 3 (cells were from 3 rats). The data were normalized by NC and shown as mean ± SE, analyzed by Student's *t*‐test, ****p < 0.0001. (C, D) GIP treatment resulted in enhanced activation of Akt. The phosphorylation of both Ser473 and Thr308 site was increased. *n* = 3. The data were normalized by NC and shown as mean ± SE, analyzed by Student's *t*‐test, ***p < 0.001. (E, F) Akt inhibitor attenuated GIP‐induced upregulation of GLUT3. *n* = 3. The data were shown as mean ± SE, analyzed by one‐way ANOVA, *p < 0.05, **p < 0.01.

GIP increases glucose uptake in neurons, the raised question is whether GIP promotes glucose uptake by affecting the expression of glucose transporters. We examined the expression levels of glucose transporters upon GIP treatment in neurons. The cellular immunofluorescence results showed that GIP could upregulate the expression level of glucose transporter GLUT3 on the neuronal cell membrane (Figure 7A,B), while did not significantly affect the other two glucose transporters, GLUT1 and GLUT4 (Figure S5). Western blots also showed that GIP up‐regulated the protein expression level of the glucose transporter GLUT3 (Figure 7E,F).

Since GIP increases the expression level of the neuron‐specific glucose transporter GLUT3 and increases the glucose uptake of neurons, we further investigated what pathway is involved in the GIP‐mediated upregulation of GLUT3. We examined some signal molecules associated with GIP/GIPR pathway including Akt, mTOR, PKA, CREB, and AMPK. Western blots showed that the level of phosphorylated Akt significantly up‐regulated upon GIP treatment (Figure 7C,D). Then the Akt‐specific inhibitor MK2206 was administrated to investigate the correlation between GIP and GLUT3, and the results (Figure 7E,F) showed that MK2206 attenuated the GIP‐mediated upregulation of GLUT3. This suggests that GIP might regulate glucose uptake by up‐regulating GLUT3 expression through Akt pathway. It has been shown that activation of Akt can enable glucose uptake by promoting GLUT1 accumulation at the plasma membrane.^26^ Whereas in our study, we found that the GIP‐induced upregulation of GLUT3 level was significantly inhibited using the Akt pathway inhibitor MK2206, which suggests that GIP increases the expression level of GLUT3 in an Akt pathway‐dependent manner. As shown in Figure S6, GIP does not show a significant effect on the activation of mTOR, PKA, CREB, and AMPK signaling pathways.

### 3.7 GIP increased GLUT3 expression via up‐regulating the level of HIF‐1α in neurons

**FIGURE 8:**
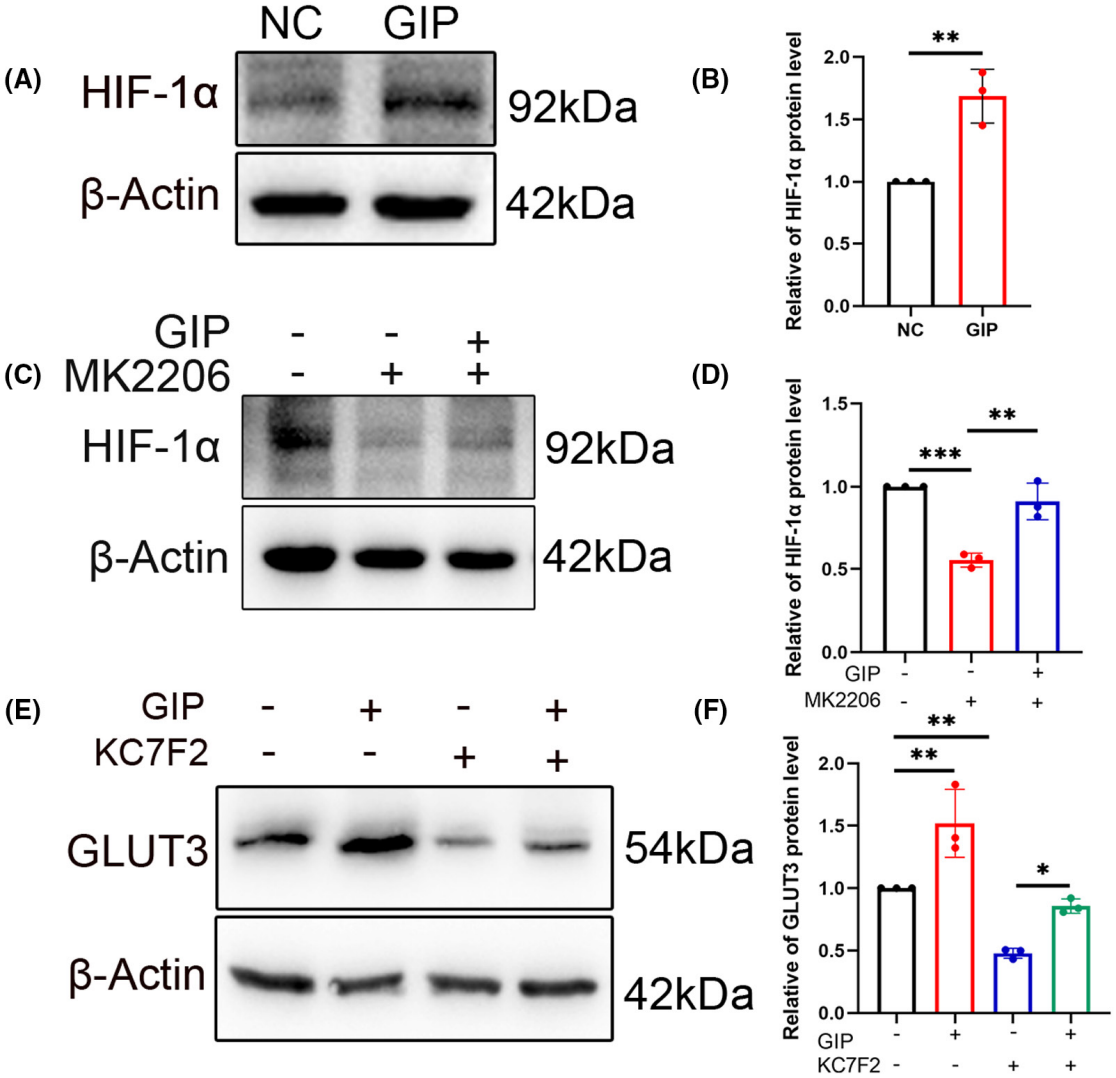
GIP increased GLUT3 expression via HIF‐1α/Akt pathways in neurons. (A, B) GIP treatment increased HIF‐1α level. *n* = 3. The data were shown as mean ± SE, analyzed by Student's *t*‐test, **p < 0.01. (C, D) Akt inhibitor MK2206 reduced HIF‐1α level both in the presence and absence of GIP. *n* = 3. The data were shown as mean ± SE, analyzed by one‐way ANOVA, **p < 0.01, ***p < 0.001. (E, F) HIF‐1α inhibitor attenuated GIP‐induced upregulation of GLUT3. *n* = 3. The data were shown as mean ± SE, analyzed by one‐way ANOVA. *p < 0.05, **p < 0.01.

The transport of glucose across cell membranes is mainly facilitated by glucose transporter 3 (GLUT3). In ischemia/reperfusion injured brains, an increase of IGF‐1 secretion and GLUT3 upregulation, are regarded as protective processes. Recent works have shown that various growth factors and cytokines including IGF‐1 can stimulate HIF‐1α expression, thereby triggering transcription of numerous hypoxia‐inducible genes by oxygen‐independent mechanisms.^27^ So, we hypothesized that HIF‐1α might play an important role in the process of GIP‐induced GLUT3.To examine the effect of GIP on HIF‐1α and GLUT3 expression, we measured their protein levels in GIP‐stimulated neurons. The protein was analyzed by western blot using antibodies to HIF‐1α and GLUT3. We observed that GIP increased the protein level of both HIF‐1α and GLUT3 (Figure 8A,B,E,F).

In addition, the Akt‐specific inhibitor MK2206 was administrated to investigate the correlation between GIP and HIF‐1α, and the results showed that MK2206 can reduce the expression level of HIF‐1α and attenuate the GIP‐mediated upregulation of HIF‐1α. This suggests that GIP might regulate glucose uptake by up‐regulating HIF‐1α expression through Akt pathway (Figure 8C,D).

Then, we investigated whether the level of HIF‐1α could affect the GLUT3 expression. KC7F2 is a potent HIF‐1α pathway inhibitor. Western blots showed that KC7F2 could reduce the expression level of GLUT3 and attenuate the GIP‐mediated upregulation of GLUT3. This suggested that GIP might regulate glucose uptake by up‐regulating GLUT3 expression via HIF‐1α pathway (Figure 8E,F). The transcription factor HIF‐1α has been reported to bind the promoter of GLUT3 gene and increase its transcription,^28^, ^29^ which is consistent with the effect of GIP in our study. In summary, we have identified a novel mechanism through the above studies: GIP‐Akt‐ HIF‐1α‐GLUT3 signaling axis, which regulates glucose transporters in neurons.

## 4 DISCUSSION

GIP/GIPR signaling exerts multiple physiological and pathological functions beyond a gastrointestinal incretin. GIP can also be detected in the cerebral cortex, and neurons could produce or respond to GIP signals.^30^, ^31^ Previous studies have shown that GIP promotes axonal growth of cortical neurons.^9^ In this study, we investigated the effect of GIP on neurons undergoing oxidative stress. The results showed that GIP could decrease ROS level in neurons after oxidative damage. This effect was further verified in vivo using a rat model of spinal cord hemisection injury.

The antioxidant effect of GIP had been proposed by previous studies,^24^, ^32^ while the underlying mechanisms were not well documented. In this study, we revealed that GIP could facilitate glucose intake in an insulin‐independent manner, and the antioxidant effect was significantly attenuated when the GIP‐enhanced glucose uptake was inhibited. Glucose metabolism and oxidative stress are two important metabolic processes in organisms. We examined the expression of some glycolytic genes after oxidative stress in neurons and found that oxidative stress inhibited the expression of glycolytic genes in neurons, while GIP stimulation reversed this effect. These results indicated that an augmented glycolysis occurred upon GIP treatment, which might contribute to the antioxidant effect.

We demonstrated that GIP might promote glycolysis and alleviate oxidative stress by increasing glucose transport. Therefore, we examined the expression of glucose transporters that are present in neuronal cells.^33^ The results showed that GIP could increase the expression of GLUT3, a neuron‐specific glucose transporter, by activating Akt pathway. The transport and utilization of glucose ensures the substantial amount of energy required for neuronal activity.^34^ Following neuronal injury, energy fluctuations induced by changes in glucose uptake are strongly associated with neuronal survival.^35^ To explicit the mechanism of how GIP increases GLUT3 level, we screened the expression of some transcription factors and found that GIP could increase the level of HIF‐1α, which is a key regulator of cell metabolism against ischemia or oxidative stress.^36^, ^37^ HIF‐1α promoted glycolysis and inhibited oxidative phosphorylation for the energy metabolism.^38^, ^39^ Then, we could speculate that GIP exerted the antioxidant effect by augmenting the glycolytic process while inhibiting oxidative phosphorylation during glucose metabolism. We further reported the activation of Akt was essential for HIF‐1α upregulation. PI3K/Akt is required for heat shock proteins to stabilize HIF‐1α.^40^ Then, we linked the GIP signal to the level of HIF‐1α, which could subsequently affect glucose transport and metabolism.

In this study, we demonstrated the beneficial effect of GIP on the recovery of spinal cord injury. The expression of GIP and GIPR after spinal cord injury had been described previously,^41^ and our results showed the exogenous administration of GIP facilitated wound healing and decreased the number of apoptotic cells, and this effect might be due to the protective effect of GIP for the neurons adjacent to epicenter of injury site. In addition, the results showed that more neuronal projections were presented in the injury sites after the treatment of GIP, which might be due to the stimulative effect on axon growth. These effects have been described in our previous study.^9^, ^10^ Then, we could speculate a promising approach for the application of GIP or GIPR agonist in spinal cord injury, which could combine the properties of neuronal protection and axon regeneration. Moreover, GIP and GIPR agonists are feasible for crossing the blood–brain barrier,^42^, ^43^ which is another advantage for their potential application. In conclusion, our study provides a mechanism insight into the protective effect of GIP on neurons under oxidative stress, meanwhile, our data also proposes an alternative approach for the treatment of spinal cord injury.

## AUTHOR CONTRIBUTIONS

Yan Liu, Mei Liu and Tuchen Guan, conceptualization and designing project; Beibei Guo, Xiaoqian Luo, Man Xu, and Yufang Zhang methodology; Mengwei Qi, Beibei Guo, Longyu Guo, Mingxuan Li, Zhen Li, and Ronghua, Wu formal analysis; Yan Liu, Beibei Guo, Mengwei Qi, Tuchen Guan writing; Yan Liu and Mei Liu supervision and administration.

## CONFLICT OF INTEREST STATEMENT

The authors declared that they have no conflicts of interest with the contents of this article.

## Supporting information


Figure S1.

## Data Availability

The data supporting the findings in this study are available within the article and supplemental information.

## References

[1] Wang HL , Zheng ZL , Han W , et al. Metformin promotes axon regeneration after spinal cord injury through inhibiting oxidative stress and stabilizing microtubule. Oxidative Med Cell Longev. 2020;2020:1‐20. 10.1155/2020/9741369 PMC6969994 31998447

[2] Wang JY , Chen P , Han GJ , et al. Rab32 facilitates Schwann cell pyroptosis in rats following peripheral nerve injury by elevating ROS levels. J Transl Med. 2024;22(1):194. 38388913 10.1186/s12967-024-04999-x PMC10885539

[3] Venditti P , Di Meo S . The role of reactive oxygen species in the life cycle of the mitochondrion. Int J Mol Sci. 2020;21(6):2173. 32245255 10.3390/ijms21062173 PMC7139706

[4] Simmons EC , Scholpa NE , Schnellmann RG . Mitochondrial biogenesis as a therapeutic target for traumatic and neurodegenerative CNS diseases. Exp Neurol. 2020;329:113309. 32289315 10.1016/j.expneurol.2020.113309 PMC7735537

[5] Gasbjerg LS , Gabe MBN , Hartmann B , et al. Glucose‐dependent insulinotropic polypeptide (GIP) receptor antagonists as anti‐diabetic agents. Peptides. 2018;100:173‐181. 29412817 10.1016/j.peptides.2017.11.021

[6] Holst JJ , Rosenkilde MM . Recent advances of GIP and future horizons. Peptides. 2020;125:170230. 31838219 10.1016/j.peptides.2019.170230

[7] Ahlqvist E , Osmark P , Kuulasmaa T , et al. Link between GIP and osteopontin in adipose tissue and insulin resistance. Diabetes. 2013;62(6):2088‐2094. 23349498 10.2337/db12-0976 PMC3661641

[8] Buhren BA , Gasis M , Thorens B , Müller HW , Bosse F . Glucose‐dependent insulinotropic polypeptide (GIP) and its receptor (GIPR): cellular localization, lesion‐affected expression, and impaired regenerative axonal growth. J Neurosci Res. 2009;87(8):1858‐1870. 19170165 10.1002/jnr.22001

[9] Teng L , Guan T , Guo B , et al. GIP‐GIPR promotes neurite outgrowth of cortical neurons in Akt dependent manner. Biochem Biophys Res Commun. 2021;534:121‐127. 33321289 10.1016/j.bbrc.2020.11.120

[10] Guan T , Guo B , Zhang W , et al. The activation of gastric inhibitory peptide/gastric inhibitory peptide receptor axis via sonic hedgehog signaling promotes the bridging of gapped nerves in sciatic nerve injury. J Neurochem. 2023;165:842‐859. 36971732 10.1111/jnc.15816

[11] Cai HY , Yang D , Qiao J , et al. A GLP‐1/GIP dual receptor agonist DA4‐JC effectively attenuates cognitive impairment and pathology in the APP/PS1/tau model of Alzheimer's disease. J Alzheimer's Dis. 2021;83(2):799‐818. 34366339 10.3233/JAD-210256

[12] Hölscher C . Protective properties of GLP‐1 and associated peptide hormones in neurodegenerative disorders. Br J Pharmacol. 2022;179(4):695‐714. 33900631 10.1111/bph.15508 PMC8820183

[13] Wu R , Mao S , Wang Y , et al. Differential circular RNA expression profiles following spinal cord injury in rats: a temporal and experimental analysis. Front Neurosci. 2019;13:1303. 31920480 10.3389/fnins.2019.01303 PMC6916439

[14] Peng WX , Liu X , Tan CH , et al. Zinc‐α2‐glycoprotein relieved seizure‐induced neuronal glucose uptake impairment via insulin‐like growth factor 1 receptor‐regulated glucose transporter 3 expression. J Neurochem. 2021;157(3):695‐709. 33258143 10.1111/jnc.15254

[15] Ma C , Teng L , Lin G , et al. L‐leucine promotes axonal outgrowth and regeneration via mTOR activation. FASEB J. 2021;35(5):e21526. 33813773 10.1096/fj.202001798RR

[16] Niizuma K , Endo H , Chan PH . Oxidative stress and mitochondrial dysfunction as determinants of ischemic neuronal death and survival. J Neurochem. 2009;109(Suppl 1):133‐138. 19393019 10.1111/j.1471-4159.2009.05897.x PMC2679225

[17] Navarro‐Yepes J , Zavala‐Flores L , Anandhan A , et al. Antioxidant gene therapy against neuronal cell death. Pharmacol Ther. 2014;142(2):206‐230. 24333264 10.1016/j.pharmthera.2013.12.007 PMC3959583

[18] Van Der Pol A , Van Gilst WH , Voors AA , et al. Treating oxidative stress in heart failure: past, present and future. Eur J Heart Fail. 2019;21(4):425‐435. 30338885 10.1002/ejhf.1320 PMC6607515

[19] Gu C , Li L , Huang Y , et al. Salidroside ameliorates mitochondria‐dependent neuronal apoptosis after spinal cord ischemia‐reperfusion injury partially through inhibiting oxidative stress and promoting Mitophagy. Oxidative Med Cell Longev. 2020;2020:3549704. 10.1155/2020/3549704 PMC7396093 32774670

[20] Halladin NL . Oxidative and inflammatory biomarkers of ischemia and reperfusion injuries. Dan Med J. 2015;62(4):B5054. 25872540

[21] Holzerová E , Prokisch H . Mitochondria: much ado about nothing? How dangerous is reactive oxygen species production? Int J Biochem Cell Biol. 2015;63:16‐20. 25666559 10.1016/j.biocel.2015.01.021 PMC4444525

[22] Yang S , Li H , Tang L , et al. Apelin‐13 protects the heart against ischemia‐reperfusion injury through the RISK‐GSK‐3β‐mPTP pathway. Arch Med Sci. 2015;11(5):1065‐1073. 26528352 10.5114/aoms.2015.54863 PMC4624751

[23] Tschöp M , Nogueiras R , Ahrén B . Gut hormone‐based pharmacology: novel formulations and future possibilities for metabolic disease therapy. Diabetologia. 2023;66:1796‐1808. 37209227 10.1007/s00125-023-05929-0 PMC10474213

[24] Verma MK , Goel R , Nandakumar K , Nemmani KVS . Effect of D‐ala(2)GIP, a stable GIP receptor agonist on MPTP‐induced neuronal impairments in mice. Eur J Pharmacol. 2017;804:38‐45. 28366809 10.1016/j.ejphar.2017.03.059

[25] Zheng J , Xie Y , Ren L , et al. GLP‐1 improves the supportive ability of astrocytes to neurons by promoting aerobic glycolysis in Alzheimer's disease. Mol Metab. 2021;47:101180. 33556642 10.1016/j.molmet.2021.101180 PMC7905479

[26] Hoxhaj G , Manning BD . The PI3K‐AKT network at the interface of oncogenic signalling and cancer metabolism. Nat Rev Cancer. 2020;20(2):74‐88. 31686003 10.1038/s41568-019-0216-7 PMC7314312

[27] Yu J , Li JL , Zhang SY , et al. IGF‐1 induces hypoxia‐inducible factor 1 alpha‐mediated GLUT3 expression through PI3K/Akt/mTOR dependent pathways in PC12 cells. Brain Res. 2012;1430:18‐24. 22104347 10.1016/j.brainres.2011.10.046

[28] Lauer V , Grampp S , Platt J , et al. Hypoxia drives glucose transporter 3 expression through hypoxia‐inducible transcription factor (HIF)‐mediated induction of the long noncoding RNA NICI. J Biol Chem. 2020;295(13):4065‐4078. 31690629 10.1074/jbc.RA119.009827 PMC7105321

[29] Mimura I , Nangaku M , Kanki Y , et al. Dynamic change of chromatin conformation in response to hypoxia enhances the expression of GLUT3 (SLC2A3) by cooperative interaction of hypoxia‐inducible factor 1 and KDM3A. Mol Cell Biol. 2012;32(15):3018‐3032. 22645302 10.1128/MCB.06643-11 PMC3434521

[30] Figueiredo CP , Antunes VLS , Moreira ELG , et al. Glucose‐dependent insulinotropic peptide receptor expression in the hippocampus and neocortex of mesial temporal lobe epilepsy patients and rats undergoing pilocarpine induced. Peptides. 2011;32(4):781‐789. 21185343 10.1016/j.peptides.2010.12.010

[31] Nyberg J , Jacobsson C , Anderson MF , Eriksson PS . Immunohistochemical distribution of glucose‐dependent insulinotropic polypeptide in the adult rat brain. J Neurosci Res. 2007;85(10):2099‐2119. 17510976 10.1002/jnr.21349

[32] Ojima A , Matsui T , Maeda S , Takeuchi M , Yamagishi S . Glucose‐dependent insulinotropic polypeptide (GIP) inhibits signaling pathways of advanced glycation end products (AGEs) in endothelial cells via its antioxidative properties. Horm Metab Res. 2012;44(7):501‐505. 22581648 10.1055/s-0032-1312595

[33] Liu CC , Hu J , Tsai CW , et al. Neuronal LRP1 regulates glucose metabolism and insulin signaling in the brain. J Neurosci. 2015;35(14):5851‐5859. 25855193 10.1523/JNEUROSCI.5180-14.2015 PMC4388937

[34] Scholpa NE , Schnellmann RG . Mitochondrial‐based therapeutics for the treatment of spinal cord injury: mitochondrial biogenesis as a potential pharmacological target. J Pharmacol Exp Ther. 2017;363(3):303‐313. 28935700 10.1124/jpet.117.244806 PMC5676296

[35] Zhang JQ , Wu PF , Long LH , et al. Resveratrol promotes cellular glucose utilization in primary cultured cortical neurons via calcium‐dependent signaling pathway. J Nutr Biochem. 2013;24(4):629‐637. 22819552 10.1016/j.jnutbio.2012.02.015

[36] Li X , Zhang Q , Nasser MI , et al. Oxygen homeostasis and cardiovascular disease: a role for HIF? Biomed Pharmacother. 2020;128:110338. 32526454 10.1016/j.biopha.2020.110338

[37] Pan Z , Ma G , Kong L , du G . Hypoxia‐inducible factor‐1: regulatory mechanisms and drug development in stroke. Pharmacol Res. 2021;170:105742. 34182129 10.1016/j.phrs.2021.105742

[38] Miska J , Lee‐Chang C , Rashidi A , et al. HIF‐1α is a metabolic switch between glycolytic‐driven migration and oxidative phosphorylation‐driven immunosuppression of Tregs in glioblastoma. Cell Rep. 2022;39(10):110934. 35675772 10.1016/j.celrep.2022.110934

[39] Williams KJ , Telfer BA , Airley RE , et al. A protective role for HIF‐1 in response to redox manipulation and glucose deprivation: implications for tumorigenesis. Oncogene. 2002;21(2):282‐290. 11803471 10.1038/sj.onc.1205047

[40] Zhou J , Schmid T , Frank R , Brüne B . PI3K/Akt is required for heat shock proteins to protect hypoxia‐inducible factor 1alpha from pVHL‐independent degradation. J Biol Chem. 2004;279(14):13506‐13513. 14726529 10.1074/jbc.M310164200

[41] Marcos AB , Forner S , Martini AC , et al. Temporal and regional expression of glucose‐dependent Insulinotropic peptide and its receptor in spinal cord injured rats. J Neurotrauma. 2016;33(3):261‐268. 26421658 10.1089/neu.2015.3877 PMC4744885

[42] Hölscher C . The incretin hormones glucagonlike peptide 1 and glucose‐dependent insulinotropic polypeptide are neuroprotective in mouse models of Alzheimer's disease. Alzheimers Dement. 2014;10(1 Suppl):S47‐S54. 24529525 10.1016/j.jalz.2013.12.009

[43] Ji C , Xue GF , Li G , Li D , Hölscher C . Neuroprotective effects of glucose‐dependent insulinotropic polypeptide in Alzheimer's disease. Rev Neurosci. 2016;27(1):61‐70. 26351802 10.1515/revneuro-2015-0021

