# Supplementary material for: GIP attenuates neuronal oxidative stress by regulating glucose uptake in spinal cord injury of rat
Source: CNS Neurosci Ther. 2024 Jun 17;30(6):e14806. doi: 10.1111/cns.14806 (PMC11183929; doi:10.1111/cns.14806)
Supplement: Supplementary file 1 — Figure S1. [file CNS-30-e14806-s001.docx]

**Supplementary Materials**


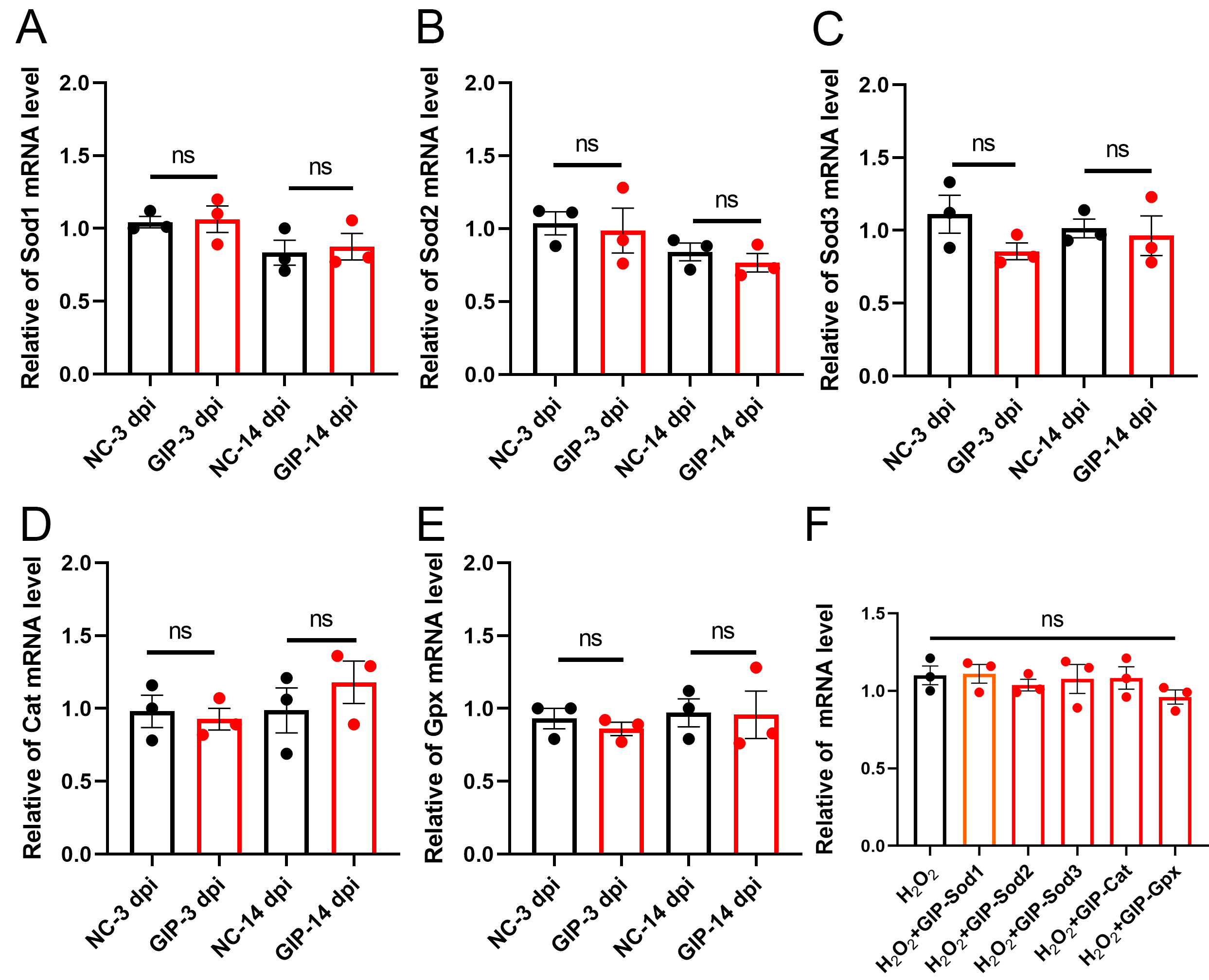


Supplementary Figure 1.GIP does not affect mRNA of antioxidant enzymes. A-E: Sod1, Sod2, Sod3, Cat, and Gpx mRNA level upon GIP treatment in tissues at 3 and 14 days after spinal cord injury. n=3. The data were shown as mean ± SE, and were analyzed by Student’s t-test. ns represents no statistical difference. F: Sod1, Sod2, Sod3, Cat, and Gpx mRNA level upon GIP treatment in primary culture. n=3. The data were shown as mean ± SE, and were analyzed by by one way ANOVA. ns represents no statistical difference.


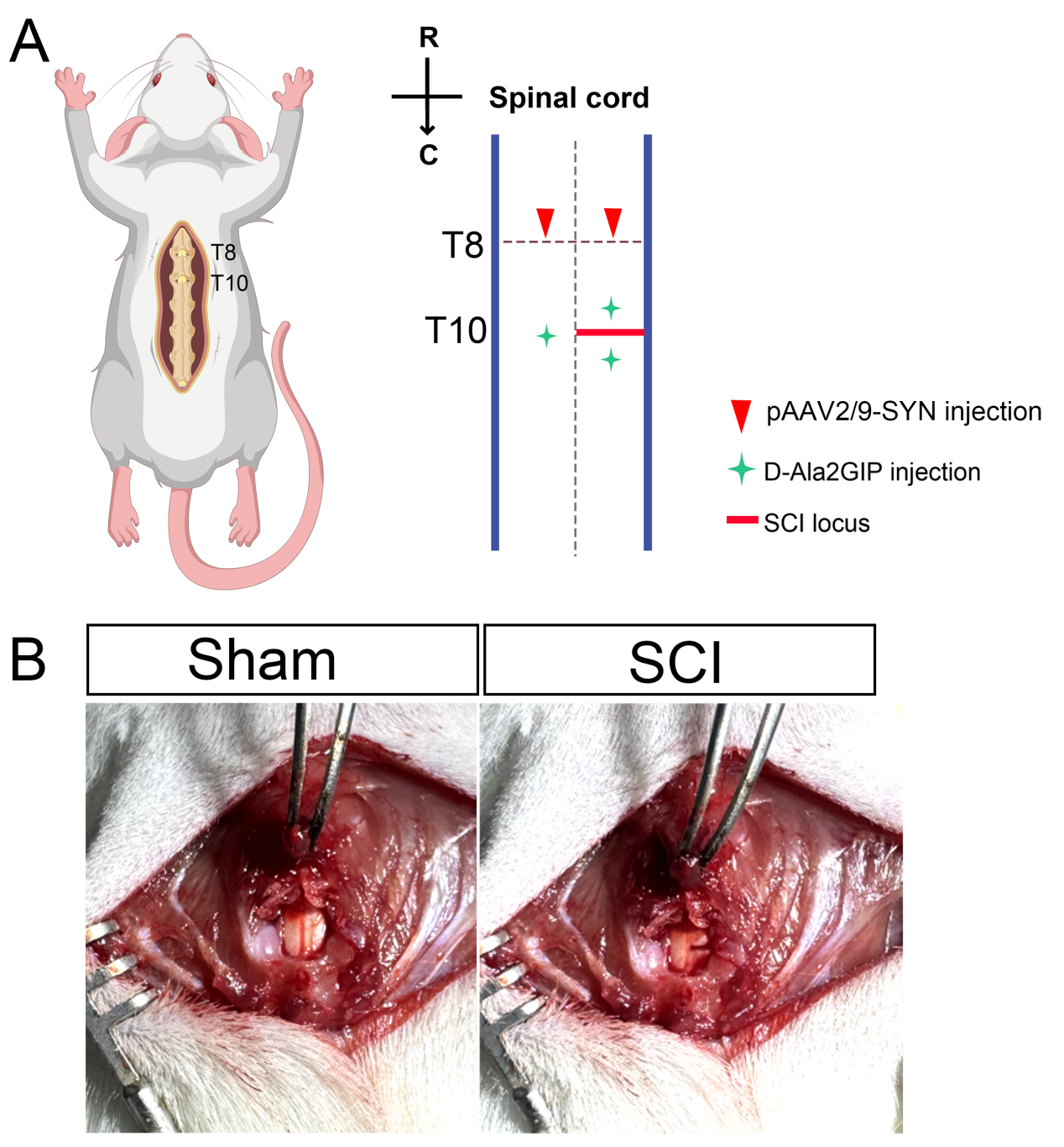


Supplementary Figure 2. Schematic diagram of spinal cord hemi-section injury. A: The position of spinal cord hemi-section, as well as the position of tracer virus and GIP injection sites. B: Spinal cord hemi-section injury surgical diagram.


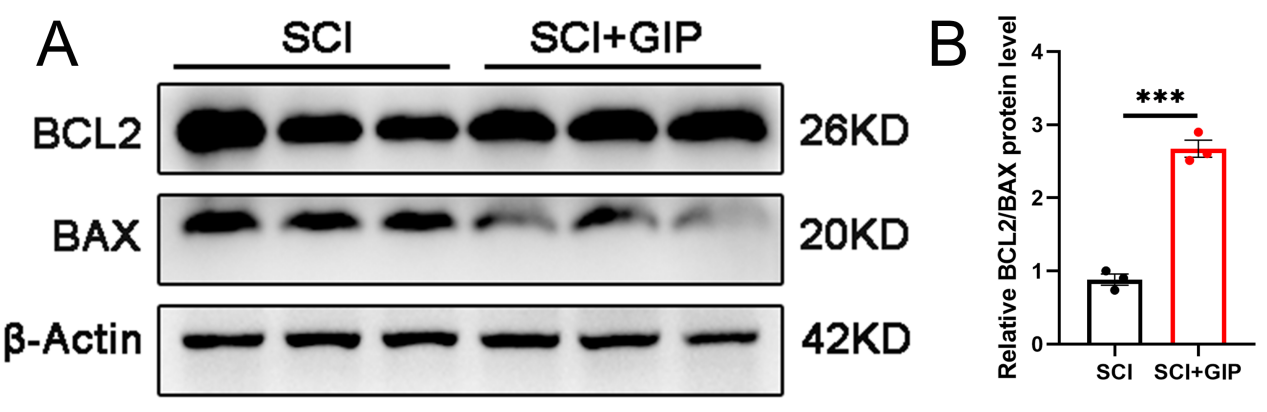


Supplementary Figure 3. GIP inhibits protein levels of BAX after spinal cord injury. A-B: Representative image and statistical result of BCL2 and BAX level in spinal cord injury at 3 dpi upon GIP treatment. n=3. The data were shown as mean ± SE, and were analyzed by Student’s t-test. ***P<0.001.


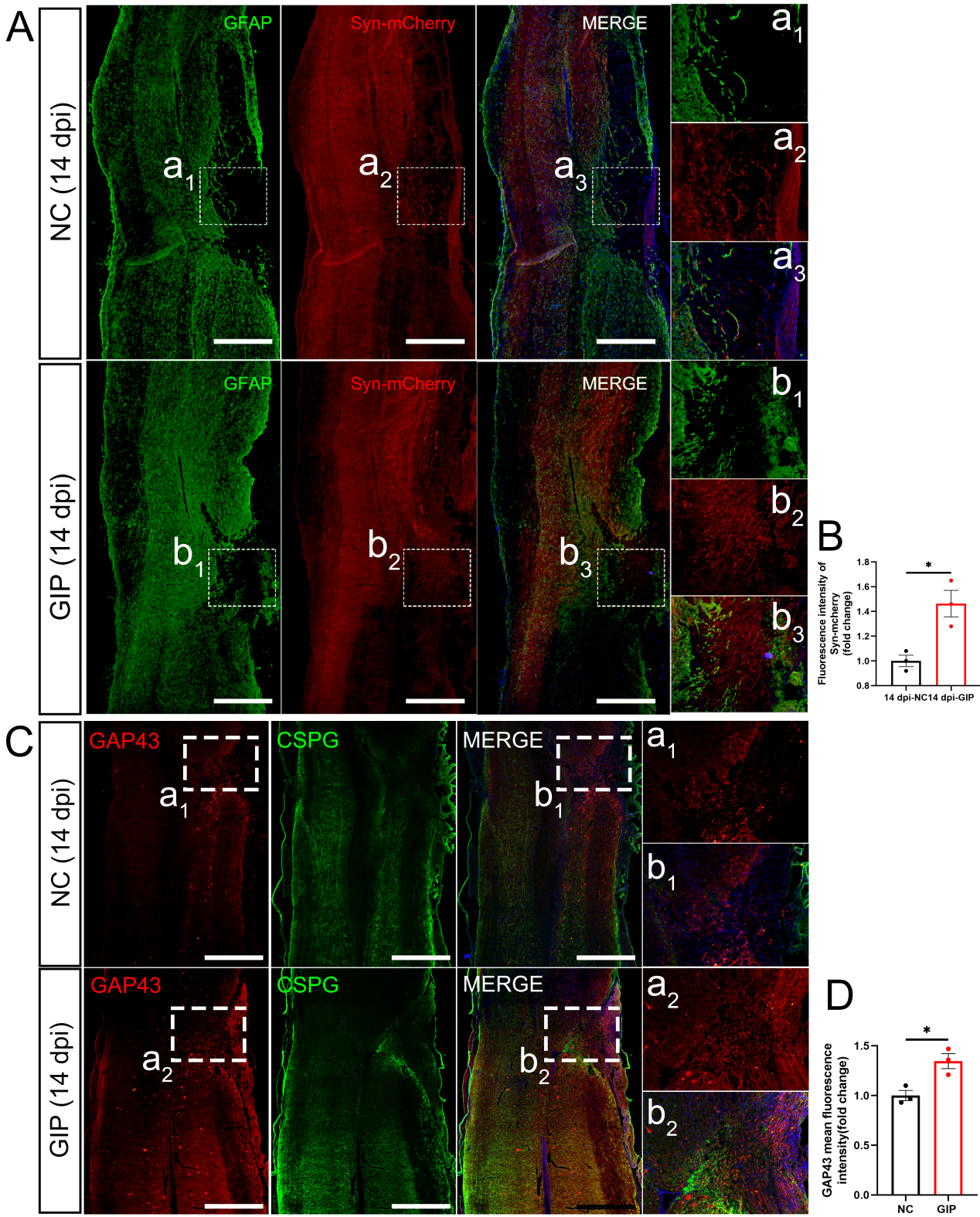


Supplementary Figure 4. GIP promotes axonal growth after spinal cord injury. A-B: Representative immunostaining staining and statistical results of Syn-mCherry (in Red), GFAP (in Green) at 14 dpi. Spinal cord was treated with NC or D-Ala^2^GIP. Propriospinal neurons were label with mCherry. The white dotted area shows the lesion zone, on the right is the enlarged views of the area within the white dashed box. Scale bar=750 μm. n=3. The data were shown as mean ± SE, and were analyzed by Student’s t-test. *P<0.05. C-D: Representative immunostaining staining and statistical results of GAP43 (in Red), CSPG (in Green) at 14 dpi. Spinal cord was treated with NC or D-Ala^2^GIP. Neurons were label with GAP43. The white dotted area shows the lesion zone, on the right is the enlarged views of the area within the white dashed box. Scale bar=750 μm. n=3. The data were shown as mean ± SE, and were analyzed by Student’s t-test. *P<0.05.


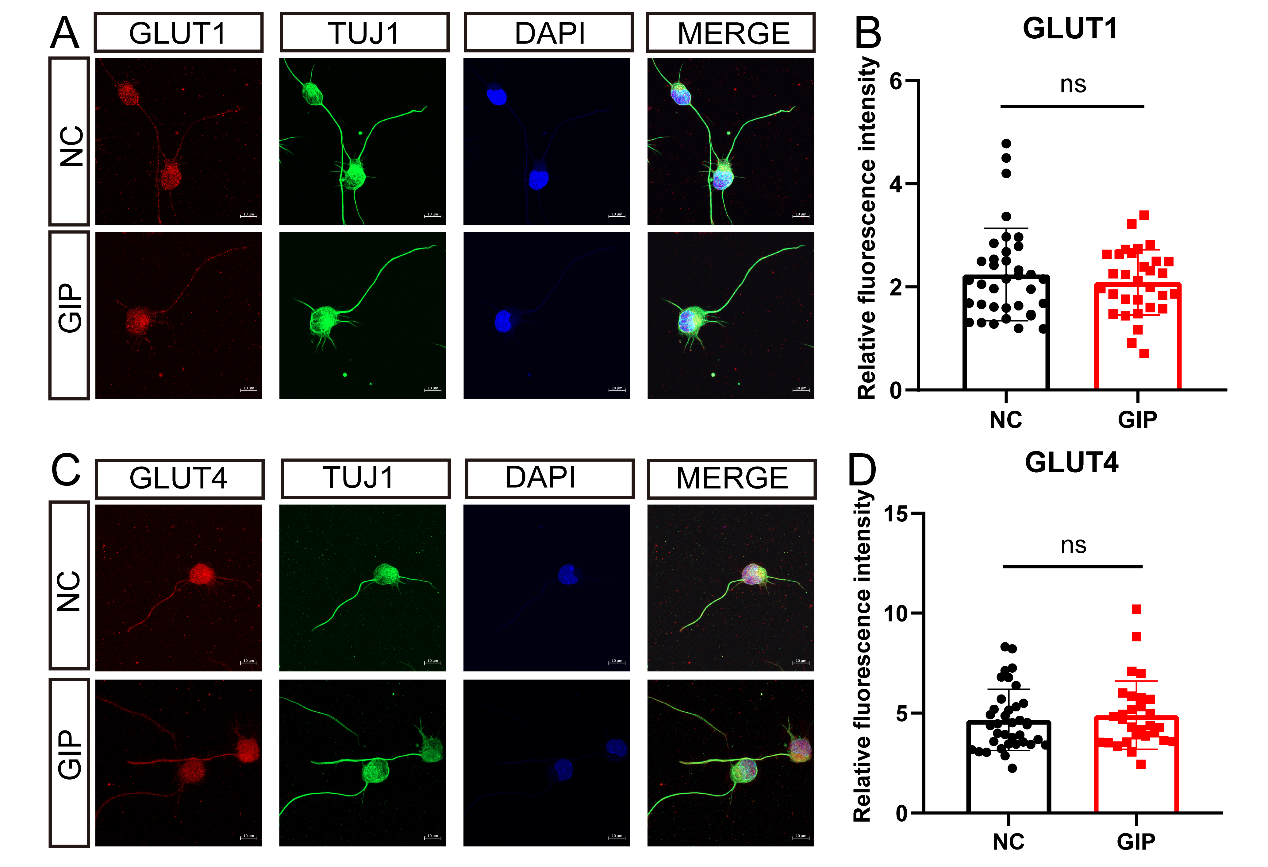


Supplementary Figure 5. The effect of GIP on GLUT1 and GLUT4 in cultured neurons. A-B: Representative images and statistical results of GLUT1 level in cortical neurons upon GIP treatment. GLUT1 (Red), TUJ1 (Green), DAPI (Blue). Scale Bar=10 μm. n≥20 neurons were included for each group per test, the experiment repeated 3 times independently. C-D: Representative images and statistical results of GLUT4 level in cortical neurons upon GIP treatment. GLUT4 (Red), TUJ1 (Green), DAPI (Blue). Scale Bar=10 μm. n≥20 neurons were included for each group per test, N=3 (cells were from 3 rats). The data were shown as mean ± SE, and were analyzed by Student’s t-test.


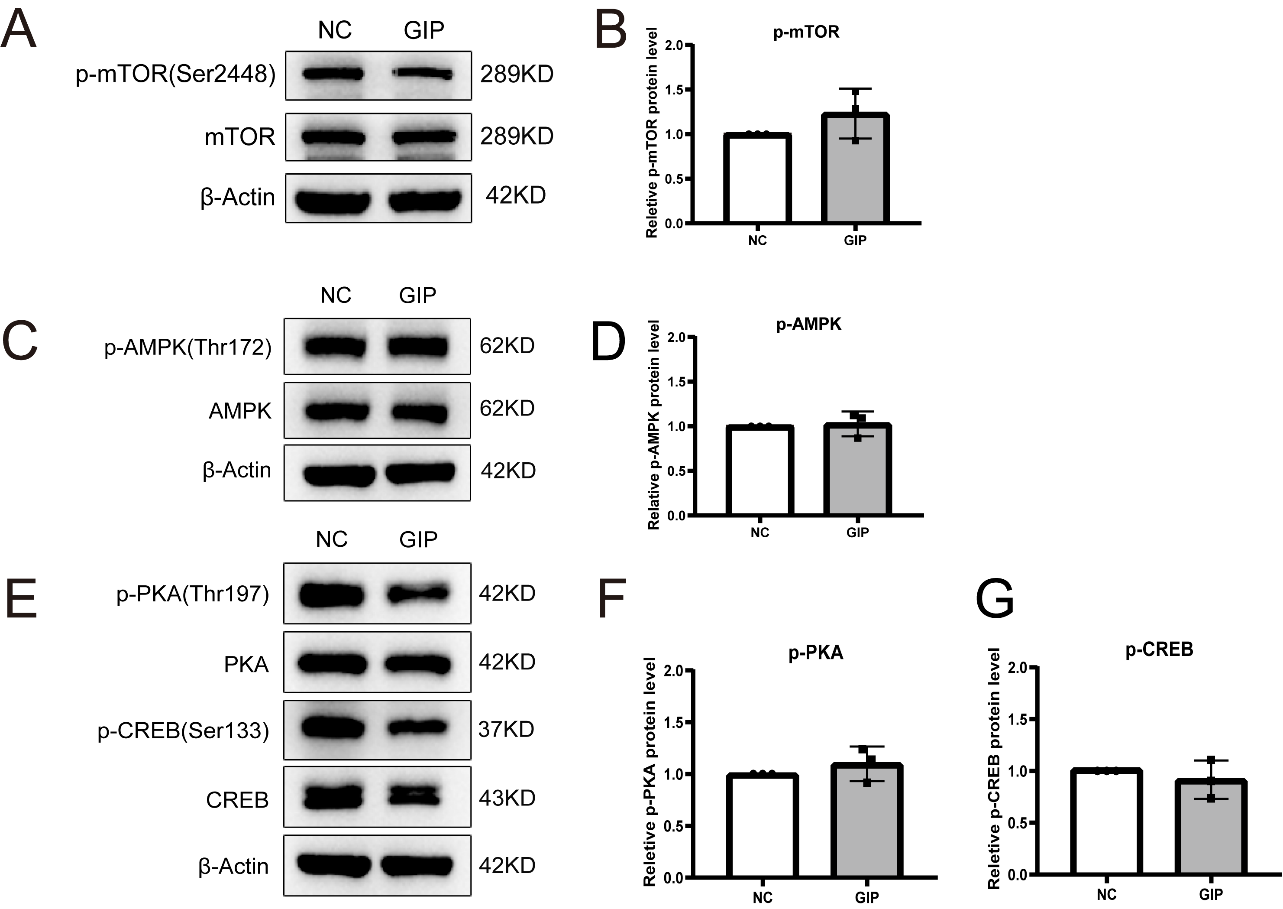


Supplementary Figure 6. Effects of GIP on activation of mTOR, AMPK, and PKA pathway in cultured neurons. A-B: Effects of GIP on phosphorylation of mTOR. n=3. The data were shown as mean ± SE, and were analyzed by Student’s t-test. C-D: Effects of GIP on phosphorylation of AMPK. n=3. The data were shown as mean ± SE, and were analyzed by Student’s t-test. E-G: Effects of GIP on phosphorylation of PKA and CREB. n=3. The data were shown as mean ± SE, and were analyzed by Student’s t-test.

­­
